# The Cytosolic Oligosaccharide-Degrading Proteome of *Butyrivibrio Proteoclasticus*

**DOI:** 10.3390/proteomes3040347

**Published:** 2015-10-27

**Authors:** Jonathan C. Dunne, William J. Kelly, Sinead C. Leahy, Dong Li, Judy J. Bond, Lifeng Peng, Graeme T. Attwood, T. William Jordan

**Affiliations:** 1 Rumen Microbiology, Animal Science Group, AgResearch Limited, Grasslands Research Centre, Palmerston North 4442, New Zealand; E-Mails: jonathan.dunne@gmri.org.nz (J.C.D.); bill.kelly@agresearch.co.nz (W.J.K.); sinead.leahy@agresearch.co.nz (S.C.L.); dong.li@agresearch.co.nz (D.L.); jude.bond@dpi.nsw.gov.au (J.J.B.); graeme.attwood@agresearch.co.nz (G.T.A.); 2 Centre for Biodiscovery and School of Biological Sciences, Victoria University of Wellington, Wellington 6140, New Zealand; E-Mail: lifeng.peng@vuw.ac.nz; 3 AgResearch Limited/Victoria University of Wellington Proteomics Laboratory, Victoria University of Wellington, Wellington 6140, New Zealand

**Keywords:** *butyrivibrio proteoclasticus*, carbohydrate active enzymes, hemicellulose, oligosaccharidases, proteomics, rumen, xylan

## Abstract

The growth and productivity of ruminants depends on a complex microbial community found in their fore-stomach (rumen), which is able to breakdown plant polysaccharides and ferment the released sugars. *Butyrivibrio proteoclasticus* B316^T^ is a Gram-positive polysaccharide-degrading, butyrate-producing bacterium that is present at high numbers in the rumen of animals consuming pasture or grass silage based diets. B316^T^ is one of a small number of rumen fibrolytic microbes capable of efficiently degrading and utilizing xylan, as well as being capable of utilizing arabinose, xylose, pectin and starch. We have therefore carried out a proteomic analysis of B316^T^ to identify intracellular enzymes that are implicated in the metabolism of internalized xylan. Three hundred and ninety four proteins were identified including enzymes that have potential to metabolize assimilated products of extracellular xylan digestion. Identified enzymes included arabinosidases, esterases, an endoxylanase, and β-xylosidase. The presence of intracellular debranching enzymes indicated that some hemicellulosic side-chains may not be removed until oligosaccharides liberated by extracellular digestion have been assimilated by the cells. The results support a model of extracellular digestion of hemicellulose to oligosaccharides that are then transported to the cytoplasm for further digestion by intracellular enzymes.

## 1. Introduction

The diet of pasture fed ruminants is rich in complex structural carbohydrates which rumen microbiota are able to digest to simpler sugar structures and provide energy for the host animal. In the cell walls of monocotyledonous grasses, xylan is the second most abundant structural carbohydrate behind cellulose and consists of a β-1,4-linked xylose backbone that may be substituted with α-l-arabinofuranosyl, *O*-acetyl, and α-glucuronic or 4-*O*-methyl-d-glucuronic acid groups. The α-l-arabinofuranosyl side groups may also be esterified with ferulic and p-coumaric acids that can form covalent linkages with other xylans or with lignin [1]. The chemically heterogeneous nature of xylan necessitates the synergistic activity of a variety of extracellular polysaccharide-degrading enzymes produced by rumen microbes. Using proteomic approaches the molecular machinery required to completely dissemble these insoluble, resilient and often underutilized food sources is being revealed. These enzymes include glycoside hydrolases (GHs) and carbohydrate esterases (CEs), that with others are classified in the Carbohydrate Active Enzymes database (CAZy, http://www.cazy.org) [2].

*Butyrivibrio proteoclasticus* B316^T^ [3,4] is a polysaccharide-degrading and butyrate-producing, Gram-positive microbe that is prevalent in the rumen of pasture grazing animals or those maintained on silage based diets [4,5,6,7,8]. *B. proteoclasticus* is one of only a small number of fibrolytic rumen microbes that are able to efficiently degrade and metabolize xylan, in addition to being able to utilize arabinose, xylose, pectin and starch [3,4]. The *B. proteoclasticus* genome encodes 114 GHs that include endo-1,4-β-xylanases, β-xylosidases and α-l-arabinofuranosidases [9], therefore providing the ability to grow well on xylan *in vitro* [3]. Kelly *et al.* [9] proposed a mechanism of extracellular polysaccharide breakdown whereby a group of nine cell-associated proteins that target xylan, pectin, and starch form the core of the extracellular catalytic potential. Recent examination by two-dimensional electrophoresis (2DE) of the extracellular polysaccharide-degrading proteome of *B. proteoclasticus* supported this mechanism [10], and demonstrated that at least four (Xyn10B, Xsa43J, Pme8B, and Pel1A) of the nine extracellular enzymes are secreted when cells are grown using xylan as the only hemicellulosic carbon source. The endo-1,4-β-xylanase Xyn10B was significantly more abundant in the culture medium of xylan-grown cells, which suggests it plays a primary role in *B. proteoclasticus* mediated hemicellulose degradation. GH family 10 endoxylanases including Xyn10B are important for xylan breakdown due to their catalytic versatility, wide substrate specificity, and ability to hydrolyze heavily substituted xylooligosaccharides [11]. The homology of the Xyn10B catalytic and carbohydrate-binding domains to those in other enzymes indicates that Xyn10B is capable of liberating variable length xylooligomers from hemicellulose [12]. Additionally, the secreted xylosidase/arabinofuranosidase Xsa43J is expected to hydrolyze xylobiose, arabinoxylans and arabinogalactans. Several ATP-binding cassette (ABC) transporter substrate-binding proteins were also found to be significantly more abundant in the xylan-grown cell culture medium [10], and in the membrane proteome [13], which implies they are important for uptake of oligosaccharides derived from xylan disassembly.

Based on these results the proposed model of hemicellulose degradation and assimilation by *B. proteoclasticus* suggests extracellular hydrolysis of the xylan backbone of hemicellulose followed by transport of substituted or un-substituted xylooligosaccharides into the cell for further metabolism [10]. This model necessitates that *B. proteoclasticus* possesses the intracellular catalytic machinery that allows cells to rapidly degrade and metabolize the internalized oligosaccharides. The *B. proteoclasticus* genome encodes 90 predicted cytosolic oligosaccharide-degrading enzymes that collectively represent 38 distinct GH, CE, and glycosyl transferase (GT) families [9]. Members of the GH2, GH3, GH13, GH31, and GH43 families are the most prevalent. Notably, all of the GH2 and GH31 enzymes and most of the GH3, GH13, and GH43 enzymes are predicted to be localized to the cytosol.

We have therefore now carried out a proteomic analysis of cytosolic fractions from *B. proteoclasticus* grown on xylan or xylose with the goal of identification of intracellular carbohydrate active enzymes associated with the further metabolism of the products of extracellular digestion of polysaccharides. Two previously described proteomic workflows [10,13] were used: (1) separation of proteins by 2DE followed by use of matrix-assisted laser desorption ionization time-of-flight (MALDI-TOF and TOF/TOF) mass spectrometry for identification of protein spots excised from the gels; and (2) liquid chromatography–tandem mass spectrometry (LC-MS/MS) of tryptic digests of the cytosolic samples.

## 2. Experimental Section

### 2.1. Sample Preparation

*B. proteoclasticus* cells were grown at 37 °C under anaerobic conditions in modified DSM Medium 704 that included 0.2% yeast extract and 0.2% trypticase peptone, with either 0.1% (*w*/*v*) oat-spelt xylan or 0.5% (*w*/*v*) xylose [10,13]. Mid-log phase and stationary phase cultures were harvested at approximately OD_600_ 0.5 and OD_600_ 0.7, respectively. Cells were separated from the culture medium by centrifugation at 3000× *g* for 30 min at 4 °C. The cell pellet was then resuspended in 10 mL of ice-cold 50 mM Tris-HCl (pH 7.0) lysis buffer containing 2% (*w*/*v*) CHAPS, cOmplete^®^ Protease Inhibitor (Roche Applied Science, Mannheim, Germany), and 100 mg of Zirconia/Silica Beads (Biospec Products, Bartlesville, OK, USA) and the cells were lysed by bead-beating for 5 min using a FastPrep FP120-230 bead beater (MP Biomedicals, Santa Ana, CA, USA). The disrupted cell suspension was centrifuged at 16,000× *g* for 30 min at 4 °C to pellet the beads and cell debris. The supernatant containing the soluble cytosolic fraction was removed and stored immediately at −80 °C.

Protein extracts were purified using the phenol/methanol/ammonium acetate procedure described by Carpentier *et al.* [14]. Briefly, the cell supernatant was mixed with an equal volume of ice-cold Tris-HCl buffered phenol (pH 8.0) and vortexed vigorously for 30 min at 4 °C. The mixture was centrifuged at 8000× *g* for 5 min and the phenolic phase was removed and placed in a fresh 1.5 mL microcentrifuge tube with an equal volume of ice-cold 50 mM Tris-HCl (pH 7.0). The mixture was vigorously vortexed for 30 min at 4 °C and then centrifuged at 8000× *g* for 5 min and the phenol phase removed and placed in a fresh 1.5 mL microcentrifuge tube. Proteins were precipitated by the addition of 4 vols of −20 °C methanol containing 100 mM ammonium acetate. The mixture was vigorously vortexed for 60 s and incubated overnight at −20 °C. The precipitated protein was pelleted by centrifugation at 13,600× *g* for 60 min at 4 °C, and the protein pellet was air-dried in a fume cupboard overnight.

### 2.2. Two-Dimensional Electrophoresis and Image Analysis

Protein samples were resuspended in 7 M urea, 2 M thiourea, 2% (*w*/*v*) CHAPS and 50 mM dithiothreitol and incubated overnight at 4 °C with shaking. Protein concentration was assayed using a 2-D Quant Kit (GE Healthcare, Uppsala, Sweden). Each sample was supplemented with 0.5% (*v*/*v*) Immobilized pH Gradient (IPG) Buffer (GE Healthcare, Uppsala, Sweden) and 125 µL was applied to 7 cm IPG strips that were passively rehydrated at room temperature for 16 h. Isoelectricfocusing (IEF), followed by second-dimension SDS-PAGE, and staining with colloidal Coomassie Brilliant Blue G250 was as previously described [10]. Briefly, IEF was for 9–11 kVh in an IPGphor (GE Healthcare, Uppsala, Sweden) followed by incubation of focused IPG strips for 15 min in 1 × LDS Buffer (Invitrogen, Carlsbad, CA, USA) with 1 × Reducing Agent (Invitrogen, Carlsbad, CA, USA), then for 15 min in LDS Sample Buffer containing 100 mM iodoacetamide. Second dimension electrophoresis was at 200 V using NuPAGE^®^ Novex 4%–12% Bis-Tris ZOOM^®^ gels in a NuPAGE^®^ MOPS Buffer (Invitrogen, Carlsbad, CA, USA). Three biological replicates were analyzed for each growth condition, and each biological replicate was analyzed in triplicate (nine gels per treatment). Stained gels were scanned using a Molecular Dynamics Personal Densitometer SI (Sunnyvale, CA, USA). Image Master™ 2D Platinum software (Version 5.0, GE Healthcare, Uppsala, Sweden) was used for gel analysis. Protein spot volumes were calculated for each spot that could be matched in at least seven of the nine analyzed replicate gels for each treatment. All detected spots were given C (cytosol fraction) numbers. A larger number of spots was detected from mid-log growth phase cells (1264 spots) compared to stationary phase cells (1180 spots). All spot volumes were within the linear range previously defined for colloidal Coomassie staining [10]. Missing data points were imputed using the lowest spot volume value for that protein detected on other gels. SPSS (Statistical Package for the Social Sciences) (Version 14.0, IBM Corporation, New York, NY, USA) software was used for statistical analysis with two-tailed Student *t*-tests (*p* < 0.01) to assess statistical significance of protein abundance changes.

### 2.3. Identification of Proteins Excised from 2DE Gels

Protein spots excised from stained 2DE gels were prepared for peptide mass fingerprinting using an Ettan Digester (GE Healthcare, Uppsala, Sweden) with 25 ng trypsin per gel plug [15]. Extracted tryptic peptides were resuspended in 1.5 µL of 10 mg.mL^−1^ α-cyano-4-hydroxycinnamic acid in 50% acetonitrile: 0.1% trifluoroacetic acid (*v*/*v*) and analyzed using a Voyager DE Pro MALDI-TOF mass spectrometer (Applied Biosystems, Foster City, CA, USA) as previously described [10,13,16]. MALDI MS/MS using an ABSCIEX 5800 TOF/TOF mass spectrometer (SCIEX, Framingham, MA, USA) was used for repeat analysis of protein spots that contained fibrolytic enzymes. TOF/TOF analysis parameters were: parent ion spectra by accumulating four sub-spectra each of 200 laser shots and spectra; then the 15 most intense ions per MS spectrum (S/N > 10) were selected for MS/MS analysis and fragment ion spectra were acquired by accumulating four sub-spectra each of 250 laser shots using air as the collision gas.

### 2.4. LC-MS/MS

Precipitated cytosolic protein extracts were resuspended in 8 M urea, 100 mM Tris-HCl (pH 8.5) at a final concentration of 0.4 µg.µL^−1^. Protein disulfide bonds were reduced with 10 mM dithiothreitol at 56 °C for 30 min, followed by alkylation with 55 mM iodoacetamide at room temperature for 40 min in the dark in the urea-buffer solution. The reduced and alkylated samples were diluted four-fold with 100 mM Tris-HCl (pH 8.5) and digested with trypsin (Roche, modified sequencing grade) at an enzyme-to-substrate ratio of 1:50 *w*/*w* with 1 mM CaCl_2_ overnight at 37 °C. Digestion was stopped by addition of formic acid to 4% (*v*/*v*) final concentration. LC-MS/MS was carried out as previously described [16] using a Finnigan LTQ™ Linear Ion-trap (Thermo Scientific, Waltham, MA, USA) with a Dionex UltiMate^®^ 3000 Nano HPLC system (Thermo Scientific, Waltham, MA, USA) containing an Acclaim^®^ PepMapTM C18 nano-column (75 µm i.d. × 15 cm, 100 Å pore size). The mobile phase gradient was constructed from 0.1% formic acid (*v*/*v*) (Solvent A) and 80% acetonitrile: 0.1% formic acid (*v*/*v*) (Solvent B). The gradient consisted of 0 to 15% linear gradient Solvent B over 25 min; 15% to 35% linear gradient Solvent B from 25 to 67 min; 35% to 100% linear gradient Buffer B from 67 to 77 min. Each biological replicate was analyzed five times to give 15 replicate raw data files per growth substrate. LC-MS/MS data files were processed using BioWorks 3.3.1 (Thermo Scientific, Waltham, MA, USA).

### 2.5. Bioinformatics

MALDI-TOF peak mass lists and MS/MS data files were searched in-house using MASCOT (Version 2.2.03) [17] against the functionally annotated *B. proteoclasticus* genome sequence database (Genbank Accession Numbers CP001810-CP001813). MASCOT search parameters were: one missed tryptic cleavage site; cysteine carbamidomethylation and methionine oxidation as fixed and variable modifications respectively; 50 ppm and 0.5 Da maximum mass tolerance for parent and fragment ions. The peptide false discovery rate (FDR) was determined by calculating the ratio of the number of unique peptides matched to the reverse protein sequences over the number of peptides matched to the reverse and forward protein sequences.

For analysis of LC-MS/MS spectra BioWorks search parameters were optimized to obtain a peptide FDR of less than 1%. Following peptide to protein assignment, the protein expectation value (less than 1.0 × 10^−4^), peptide cross correlation scores (greater than 1.5 for single charge, 2.5 for double charge and 3.3 for triple charge), and minimum number of matched peptides per protein (minimum 2) were used as filters to obtain an average protein FDR across all technical replicate search results of below 1%. Protein FDR was calculated as the ratio of the number of proteins matched to the reverse protein sequences over the number of proteins matched to the reverse and forward protein sequences, respectively. Proteins were included in the BioWorks identification dataset only if they were identified with statistical significance in at least two of the three biological replicate samples.

Protein isoelectric point and size were calculated as previously described [10]. Also as before, Pfam, Tigrfam, BLASTp and ClustalW [18,19,20,21] were used to predict protein functional domains and sequence alignments. Signal peptides were predicted using SignalP 3.0 [22], LipoP 1.0 [23] and pattern searching as recommended for Gram-positive bacteria [24]. TMHMM 2.0 [25] and SOSUI/G [26] were used to predict membrane spanning domains.

## 3. Results and Discussion

### 3.1. Theoretical Proteome

**Figure 1:**
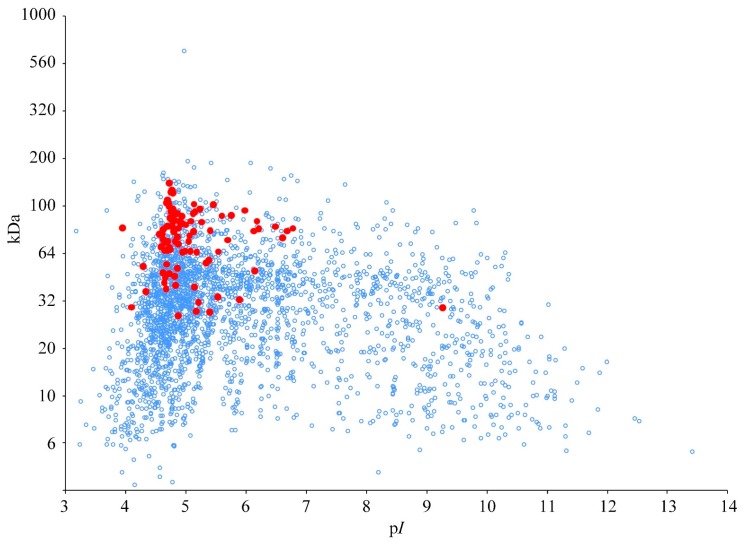
Theoretical 2DE map of the *B. proteoclasticus* cytoplasmic proteins. Red spots represent predicted oligosaccharide-degrading enzymes. Blue open circles represent all other cytoplasmic proteins. The y-axis is presented in logarithmic scale to represent separation of proteins by 2DE.

The theoretical 2DE map of the *B. proteoclasticus* intracellular cytoplasmic proteome (Figure 1) was dominated by acidic proteins: 79% (2242) of the predicted cytoplasmic proteins possessed a theoretical p*I* < 7, and 89 of the 90 predicted cytoplasmic oligosaccharide-degrading enzymes had a p*I* < 6.8 with a cluster of 81 enzymes between p*I* 4 and 5.6. Ninety percent of the predicted cytoplasmic proteome (2575 proteins) had predicted polypeptide masses between 10 and 150 kDa, and all cytoplasmic oligosaccharidases had a predicted size between 26 and 134 kDa. The group of 802 proteins with a predicted p*I* value > 6.5 was dominated by hypothetical proteins and mobile elements (transposases), as well as proteins involved in nucleic acid metabolism, protein synthesis, transcriptional regulation, and cell envelope biogenesis. Following initial 2DE using pH 3–10 IEF strips, all further analysis was carried out using narrow range focusing to enhance separation of the acidic proteins.

### 3.2. Identification of Proteins Separated by 2DE

**Figure 2:**
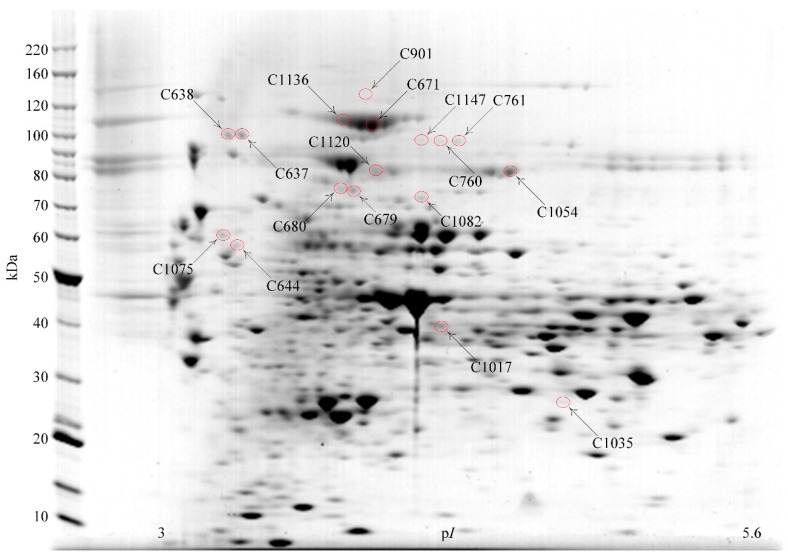
Oligosaccharide degrading enzymes identified in the *B. proteoclasticus* cytosol by 2DE/MALDI-TOF/TOF. A Coomassie-stained 2DE gel (p*I* 3–5.6) is shown of cytosolic proteins harvested from mid-log phase cells that had been grown in xylan. Circled spots indicate the identified oligosaccharidases that are summarized in Table 1.

Cytosolic subcellular fractions from cells harvested at mid-log or stationary growth phase were analyzed using 2DE of multiple gels (p*I* 3–5.6, 5.3–6.5, 6–11 IEF), followed by MALDI-TOF and MALDI-TOF/TOF analysis of tryptic digests of protein spots excised from the gels. Proteins were identified from 481 of 625 excised spots, and were the products of 219 genes. Table 1 summarizes the 17 2DE spots (Figure 2) from which 15 different enzymes that are predicted to be associated with catabolism of oligosaccharides were identified. All enzymes were identified from the p*I* 3–5.6 2DE separations. Two protein spots, C1120 and C1147, contained more than one glycosidase. C1147 contained β-galactosidase Bga2B, β-glucosidase Bgl3C and β-mannosidase Man2A. The predicted p*I* and size of the three enzymes were similar. Spot C1120 contained β-galactosidase Bga35B and β-xylosidase Xyl3A, both of which were also of similar predicted p*I* and size. Other identified proteins are listed in Supplementary Table S1 including 28 enzymes from intracellular pathways of carbohydrate metabolism and 11 oligosaccharide ABC-transporter associated proteins.

**Table 1:** Summary of the oligosaccharidases identified by 2DE/MALDI-TOF/TOF in the *B. proteoclasticus* cytosol.

Spot	Protein	Locus	Protein Pilot Score	Expect. ^i^	SigP ^ii^	p*I*	kDa	Pep ^iii^
C901	β-Galactosidase, Bga2A	Bpr_I0279	239	4.8 × 10^−21^	N	4.7	118.7	31
C1147	β-Galactosidase, Bga2B	Bpr_III209	80	4.1 × 10^−5^	N	4.8	91.1	19
C1147	β-Mannosidase, Man2A	Bpr_III237	117	3.8 × 10^−8^	N	4.8	95.9	20
C1147	β-Glucosidase, Bgl3C	Bpr_I0138	73	1.8 × 10^−4^	N	4.9	91.5	10
C1136	β-Glucosidase, Bgl3B	Bpr_I0847	94	1.4 × 10^−6^	N	4.7	103.7	24
C671	β-Glucosidase, Bgl3B	Bpr_I0847	218	6.1 × 10^−19^	N	4.7	91.5	23
C760	β-Glucosidase, Bgl3C	Bpr_I0138	72	2.4 × 10^−4^	N	4.9	91.5	7
C761	β-Glucosidase, Bgl3C	Bpr_I0138	231	3.0 × 10^−20^	N	4.9	103.7	23
C1120	β-Xylosidase, Xyl3A	Bpr_I0184	397	7.6 × 10^−37^	N	4.8	78.2	28
C1120	β-Galactosidase, Bga35B	Bpr_I2006	87	6.9 × 10^−6^	N	4.9	83.2	12
C637	Pullulanase, Pul13A	Bpr_III161	191	3.0 × 10^−16^	Y	4.4	99.7	19
C638	Pullulanase, Pul13A	Bpr_III161	243	1.9 × 10^−21^	Y	4.4	99.7	22
C644	Glycoside hydrolase family 30, GH30A	Bpr_I2937	261	3.0 × 10^−23^	Y	4.3	67.0	23
C679	Glycoside hydrolase family 31, GH31C	Bpr_I1974	301	3.0 × 10^−27^	N	4.7	78.2	40
C680	Glycoside hydrolase family 31, GH31C	Bpr_I1974	248	6.1 × 10^−22^	N	4.7	78.2	39
C1054	α-Galactosidase, Aga36C	Bpr_III065	315	1.2 × 10^−28^	N	5.1	83.2	30
C1075	Xylosidase/arabino-furanosidase, Xsa43A	Bpr_I0302	110	3.8 × 10^−8^	Y	4.3	57.5	17
C1082	α-D-Glucuronidase, Agu67A	Bpr_I0177	341	3.0 × 10^−31^	N	4.9	76.1	32
C1035	Feruloyl esterase, Est1E	Bpr_I2870	82	2.7 × 10^−5^	N	5.2	27.8	13
C1017	Acetyl-xylan esterase, Est2A	Bpr_I2939	235	1.2 × 10^−20^	N	4.8	42.4	8

^i^ Expectation value is the statistical probability of the top ranked protein match being a false positive identification, *p* < 0.05; ^ii^ Secretory signal-peptides were predicted using SignalP (Version 3.0, Technical University of Denmark, Lyngby, Denmark), LipoP (Version 1.0, University of Denmark, Lyngby, Denmark) and pattern searching as recommended for Gram-positive bacteria [22,23,24]; ^iii^ Number of tryptic peptides matched to the full length protein sequence.

### 3.3. Protein Identification by LC-MS/MS

Tryptic digests of cytosolic extracts from xylan-grown and xylose-grown, mid-log phase cells were digested and analyzed by LC-MS/MS, using three separate cultures (biological replicates) for each condition and each biological replicate analyzed by LC MS/MS five times. A total of 329 non-redundant proteins were identified including 50 proteins involved in carbohydrate metabolism, 20 of which were oligosaccharidases (Table 2). Notably, nine of these had not been identified by the 2DE-based analyses, bringing the total number of oligosaccharidases identified in the cytosol of *B. proteoclasticus* cells to 24 (15 enzymes identified from 2D gels plus nine new identifications by LC-MS/MS). Similar to the pattern observed in the gel-based analyses, all LC-MS/MS identified enzymes had a predicted p*I* value within a narrow range between 4.1 and 6. Other identified proteins are listed in Supplementary Table S2.

**Figure 3:**
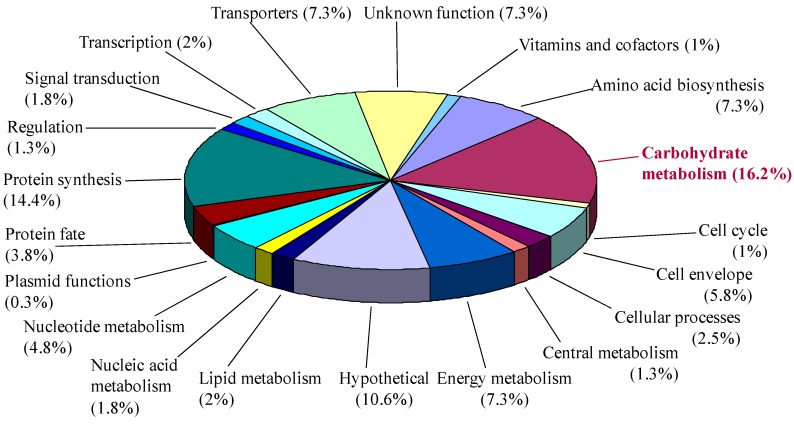
Protein function summary of all proteins identified in the *B. proteoclasticus* cytosol.

Figure 3 summarizes the functional analysis of all proteins identified either from the 2DE gels or by LC-MS/MS. Carbohydrate metabolism was the major class, comprising 16.2% of the total 394 protein identifications. In accordance with the predicted metabolic pathways of *B. proteoclasticus* [9], enzymes from the pentose phosphate pathway and enzymes involved in production of butyrate and formate were detected (Supplementary Tables S1 and S2). Other major classes included protein synthesis, transporters, amino acid biosynthesis and energy metabolism.

**Table 2:** Summary of oligosaccharide degrading enzymes identified by LC-MS/MS ^i^.

Protein	Locus	Expect. ^ii^	SigP ^iii^	p*I*	kDa	Pep ^iv^
β-Galactosidase, Bga2A	Bpr_I0279	1.5 × 10^−12^	N	4.7	118.7	8
β-Mannosidase, Man2A	Bpr_III237	3.1 × 10^−11^	N	4.8	95.9	7
β-Glucosidase, Bgl3A	Bpr_I0693	2.5 × 10^−6^	Y	4.1	115.6	23
β-Glucosidase, Bgl3B	Bpr_I0847	2.6 × 10^−11^	N	4.7	103.7	7
β-Glucosidase, Bgl3C	Bpr_I0138	8.1 × 10^−7^	N	4.9	91.5	2
β-Xylosidase Xyl3A	Bpr_I0184	8.9 × 10^−15^	N	4.8	78.2	42
Cellodextrinase, Cel9B	Bpr_I1593	1.0 × 10^−30^	N	4.6	61.0	22
Endo-1,4-β-xylanase and esterase, Xyn10D	Bpr_I1083	3.6 × 10^−10^	N	5.0	79.7	5
α-Amylase, Amy13G	Bpr_I0729	1.7 × 10^−11^	N	4.7	60.3	3
Pullulanase, Pul13A	Bpr_III161	1.1 × 10^−10^	Y	4.4	99.7	7
Glycoside hydrolase family 31, GH31C	Bpr_I1974	1.0 × 10^-11^	N	4.7	78.2	32
β-Galactosidase, Bga35B	Bpr_I2006	3.1 × 10^-10^	N	4.9	83.2	4
α-Galactosidase, Aga36C	Bpr_III065	9.3 × 10^−5^	N	5.1	83.2	7
Xylosidase/arabinofuranosidase and esterase, Xsa43H	Bpr_I0301	1.6 × 10^−8^	N	4.7	107.9	7
α-l-Arabinofuranosidase, Arf51A	Bpr_I0329	1.1 × 10^−15^	N	5.2	57.0	14
α-d-Glucuronidase, Agu67A	Bpr_I0177	4.9 × 10^−11^	N	4.9	76.1	22
α-l-Rhamnosidase, Rha78A	Bpr_I1686	99.9	N	4.8	85.0	3
Cellobiose phosphorylase, Cbp94A	Bpr_I2447	1.1 × 10^−16^	N	5.1	91.5	6
Feruloyl esterase, Est1E	Bpr_I2870	1.3 × 10^−10^	N	5.2	27.8	5
Glycogen phosphorylase, Glgp2	Bpr_I2847	1.8 × 10^−10^	N	6.0	94.8	10

^i^ Proteins shown in bold were uniquely identified by 1D LC-MS/MS; ^ii^ Expectation value is the statistical probability of the top ranked protein match being a false positive identification, *p* < 0.05; ^iii^ Secretory signal-peptides were predicted using SignalP (Version 3.0) , LipoP (Version 1.0) and pattern searching as recommended for Gram-positive bacteria [22,23,24]; ^iv^ Number of tryptic peptides matched to the full length protein sequence.

### 3.4. Functional Domains of the Oligosaccharidases

**Figure 4:**
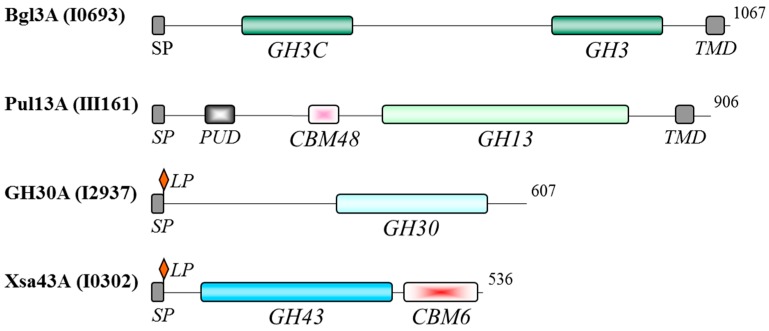
Functional domains of predicted secreted polysaccharidases identified in the *B. proteoclasticus* cytosol. Residue length is indicated to the right of each enzyme. CBM, carbohydrate-binding module; LP, lipobox motif; PUD, bacterial pullulanase-associated domain; SP, secretory signal peptide; TMD, transmembrane domain.

Pfam, BLASTp and Tigrfam analyses were used to predict carbohydrate active domains in each oligosaccharidase amino acid sequence. Four proteins, β-glucosidase Bgl3A, glycoside hydrolase GH30A, pullulanase Pul13A, and xylosidase/arabinofuranosidase Xsa43A, were each predicted to contain an N-terminal secretory signal peptide (Figure 4). Bgl3A and Pul13A also contained a predicted transmembrane helix within the C-terminal region, while GH30A and Xsa43A contained lipobox motifs at the N-terminus. It is therefore possible that Bgl3A and Pul13A are membrane-anchored proteins and that GH30A and Xsa43A are cell wall lipoproteins. Although cross-contamination of the cytosolic subcellular fraction by proteins released from the membrane or cell wall is possible, it should be noted that none of the four proteins were detected in the previously reported membrane [13] or secreted [10] fractions. Like some of the extracellular polysaccharidases [10], the potentially membrane anchored GH13 pullulanase and secreted GH43 xylosidase/arabinofuranosidase Xsa43A contained carbohydrate-binding modules (CBMs). CBM48 modules mediate enzyme attachment to α-1,4-linked glucose monomers [27], while CBM6 domains have been shown to target the parent enzyme to cellulose, xylooligosaccharides, and mixed linkage glucans [28,29]. The possible functional association of these four proteins with the membrane and cell wall remains to be assessed. Homology to the functionally characterized *Bacillus subtilis* subsp. *subtilis* ATCC 6051 XynD [30] suggests that Xsa43A may target intact or partially degraded arabinose containing xylans, hydrolysing (1→2)-α-linked and (1→3)-α-linked l-arabinofuranosyl groups from xylooligosaccharides of varying lengths. It is possible that Xsa43A augments the activity of the secreted Xsa43J [10] to hydrolyse α-linked l-arabinofuranosyl groups from the xylan backbone. GH13 pullulanases (EC 3.2.1.41) (also called debranching enzymes) hydrolyze α-1,6 glycosidic linkages in polymers such as starch, pullulan, and other branched oligosaccharides, generating α-1,4 linked linear oligosaccharides. Although the catalytic domain of GH30A is similar to the GH30 domain found within the Xsa43J [10], without functional analysis it is difficult to predict the catalytic potential of the GH30A enzyme. Functional analysis is also required to predict the catalytic activity of Bgl3A.

**Figure 5:**
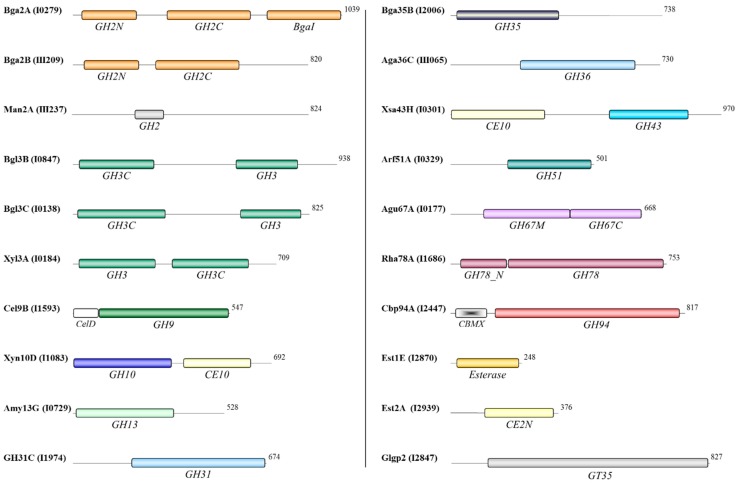
Functional domains of predicted cytoplasmic oligosaccharidases identified in the *B. proteoclasticus* cytosol.

The remaining 20 identified oligosaccharidases are predicted to be localized to the cytoplasm. This group of enzymes included three members each of the GH2 and GH3 families and single representatives of 11 other GH families (Figure 5). Two esterase families and a glycosyl transferase were also represented. Several enzymes contained more than one predicted catalytic domain, including all the GH3 family enzymes that contained GH3 and GH3C catalytic domains, and the β-galactosidase Bga2A that contained three catalytic domains. A distinguishing feature of both the GH43 xylosidase/arabinofuranosidase/esterase Xsa43H and the GH10 endo-1,4-β-xylanase/esterase Xyn10D was that each of those proteins contained two putative catalytic domains that were predicted to be active upon different substrates, namely GH and CE activities. Interestingly, among the predicted cytosolic enzymes a CBMX domain was found within the N-terminal region of the GH94 cellobiose phosphorylase, and a CelD domain is predicted in the GH9 cellodextrinase. Both non-catalytic domains are often associated with these types of enzymes, and both may play a role in enzyme/substrate attachment.

### 3.5. Effects of Growth on Xylan or Xylose

To further investigate the potential roles of carbohydrate metabolizing enzymes in the processing of xylan, 2DE was used to quantify the effects of growth substrate and growth phase on the relative abundance of fibrolytic enzymes and transport proteins. Table 3 and Supplementary Figure S1 summarize mid-log phase proteins that differed in abundance between cells grown on xylose or xylan. Three biological replicates (separate cultures) were analyzed for each substrate and growth condition, and each biological replicate was analyzed on triplicate gels. Four of the identified enzymes were only detected in cells grown on xylan. Two ABC transporter substrate-binding proteins were unique to growth on xylan. Pullulanase (Pul13A) was detected as a pair of adjacent spots at approximately p*I* 4.2 and 100 kDa, which corresponded closely with the theoretical values for this protein. The more acidic form was also only detected in cells grown on xylan, while the more basic form was 2.8 times more abundant in cells grown on xylan compared to xylose. A further eight protein spots showed greater than two-fold differences (*p* < 0.05) between growth on xylan compared to xylose, although two of these spots, C733 and C1010, each contained two identified proteins. The most strongly up- and down-regulated proteins in the mid-log phase proteome of xylan grown cells were a subtilisin family serine protease and glutamate synthase GltA, respectively.

**Table 3:** Summary of the differentially abundant proteins between xylan and xylose identified in the mid-log phase cytosolic proteome.

Spot	Protein	Locus	Function ^i^	Expect. ^ii^	p*I*	kDa	Fold-Change ^iii^	*p* Value
C853	Adenylosuccinate lyase, PurB	Bpr_I2212	O	1.2 × 10^−10^	5.4	53.7	−3.1 ± 0.6	0.001
C1135	Amino acid ABC transporter substrate-binding protein	Bpr_I1826	T	5.8 × 10^−4^	4.0	31.6	Xylan	n/a
C1010	Anti-sigma factor antagonist/phosphotransferase domain-containing protein	Bpr_I0249	R	3.0 × 10^−30^	4.6	49.8	−12.0 ± 0.7	0.001
	Ribosomal protein S1, RpsA	Bpr_I2035	Q		4.6	41.9		
C654	DNA-directed RNA polymerase α subunit, RpoA	Bpr_I0623	W	3.0 × 10^−17^	4.4	35.1	−8.7 ± 4.3	0.002
C837	Fructose-1,6-bisphosphate aldolase, FbaA	Bpr_I2903	C	3.0 × 10^−13^	5.1	30.5	−2.1 ± 0.2	0.002
C733	IMP cyclohydrolase, PurO	Bpr_I0731	O	1.5 × 10^−18^	4.7	32.2	2.3 ± 0.5	0.001
	Translation elongation factor Tu, TufA	Bpr_I2364	Q		4.82	43.6		
C792	NADPH-dependent glutamate synthase, GltA3	Bpr_I1306	A	1.9 × 10^−4^	5.0	49.3	−15.8 ± 6.3	0.001
C637	Pullulanase, Pul13A	Bpr_III161	C	1.5 × 10^−9^	4.4	99.7	2.8 ± 0.3	0.001
C638	Pullulanase, Pul13A	Bpr_III161	C	1.8 × 10−^6^	4.4	99.7	Xylan	n/a
C709	Ribosomal protein S1, RpsA	Bpr_I2035	Q	9.6 × 10^−19^	4.6	41.9	−4.1 ± 1.1	0.001
C1072	Serine protease subtilisin family	Bpr_I2629	P	3.8 × 10^−8^	3.8	153.3	5.8 ± 1.3	0.001
C1074	Sugar ABC transporter substrate-binding protein	Bpr_I0182	T	1.5 × 10^−8^	4.12	63.3	Xylan	n/a

^i^ A, Amino acid biosynthesis; C, Carbohydrate metabolism; O, Nucleotide metabolism; P, Protein fate; Q, Protein synthesis; R, Regulation; T, Transporters; W, Transcription; ^ii^ Expectation value is the statistical probability of the top ranked protein match being a false positive identification, *p* < 0.05; ^iii^ Fold-change was calculated as the ratio of the mean normalized protein spot volumes in the xylan *versus* xylose growth conditions as stated. Positive values denote proteins with increased mean normalized spot volume in xylan grown cells. “Xylan” denotes the protein was uniquely detected in culture medium harvested from xylan grown cells. In these cases it was not possible to calculate a *p* Value and “n/a” (non-applicable) is entered.

For cells harvested at stationary growth phase, two identified proteins were detected only in cells grown on xylan. They were a sugar ABC transporter substrate-binding protein (abundant spot C1074) and an amino acid ABC transporter substrate-binding protein (minor spot C1135) (Table 4, Supplementary Figure S2) that were also unique to growth on xylan in mid-log phase cells. The high abundance of the sugar transport protein Bpr_I0182 is in good agreement with its increased abundance in the membrane fraction of *B. proteoclasticus* grown with xylan compare to fructose [13]. In addition, the more basic pullulanase identified in mid-log phase xylan-grown cells was replaced by a spot of slightly lower mass that was also identified as this protein. Other identified proteins differed quantitatively between cells grown on xylan compared to xylose. Analysis of the differences was complicated by identification of two proteins in each of the spots C704, C785, and C1120. C1120 contained β-galactosidase Bga35A and β-xylosidase Xyl3A and was more abundant after growth in xylan. In contrast, the xylose ABC transporter substrate-binding protein product of Bpr_I1173 (spot C1057) was less abundant in xylan-grown cells. A U62 family peptidase identified in spot C784 (Bpr_I2456) was also more abundant in xylan grown cells. This peptidase was up to 51% identical to many clostridial zinc-dependent proteases and U62 family peptidases that modulate DNA gyrase activity [31].

**Table 4:** Summary of the differentially abundant proteins between xylan and xylose identified in the stationary phase cytosolic proteome.

Spot	Protein	Locus	Function ^i^	Expect. ^ii^	p*I*	kDa	Fold-Change ^iii^	*p* Value
C1135	Amino acid ABC transporter substrate-binding protein	Bpr_I1826	T	5.8 × 10^−4^	4.0	31.6	Xylan	n/a
C785	Aminotransferase DegT/DnrJ/EryC1/StrS family	Bpr_I2311	I	1.5 × 10^−10^	5.0	51.0	4.2 ± 1.1	0.001
	Xylulokinase, XylB	Bpr_I0173	C		4.9	53.7		
C1120	β-Galactosidase, Bga35B	Bpr_I2006	C	1.9 × 10^−27^	4.9	83.2	4.5 ± 0.8	0.001
	β-Xylosidase, Xyl3A	Bpr_I0184	C		4.8	78.2		
C704	Hypothetical protein	Bpr_I2455	H	7.6 × 10^−22^	4.7	47.7	2.3 ± 0.1	0.001
	Phosphoribosylamine-glycine ligase, PurD	Bpr_I0870	O		4.7	46.3		
C601	Oligopeptide ABC transporter substrate-binding protein, OppA1	Bpr_I1276	T	1.9 × 10^−13^	4.0	83.1	2.3 ± 0.5	0.001
C784	Peptidase U62 family	Bpr_I2456	P	6.1 × 10^−9^	4.8	52.3	14.7 ± 4.6	0.001
C1074	Sugar ABC transporter substrate-binding protein	Bpr_I0182	T	7.6 × 10^−7^	4.12	63.3	Xylan	n/a
C1057	Xylose ABC transporter substrate-binding protein	Bpr_I1173	T	9.6 × 10^−12^	4.2	38.4	−3.9 ± 1.1	0.001

^i^ C, Carbohydrate metabolism; H, Hypothetical; I, Cell envelope; O, Nucleotide metabolism; P, Protein fate; T, Transporters; ^ii^ Expectation value is the statistical probability of the top ranked protein match being a false positive identification, *p* < 0.05; ^iii^ Fold-change was calculated as the ratio of the mean normalized protein spot volumes in the xylan *versus* xylose growth conditions as stated. Positive values denote proteins with increased mean normalized spot volume in xylan grown cells. “Xylan” denotes the protein was uniquely detected in culture medium harvested from xylan grown cells. In these cases it was not possible to calculate a *p* Value and “n/a” (non-applicable) is entered.

### 3.6. Predicted Activities and Pathways

The results of this study are consistent with the model of *B. proteoclasticus* polysaccharide metabolism that has emerged from the genomic [9], and proteomic analyses of the extracellular [10] and membrane compartments [13]. This model proposes that a core set of secreted enzymes target primarily the xylan backbone and are therefore likely to liberate a range of substituted or unsubstituted xylooligosaccharides. One caution is that gene identifications were based on homology with other bacterial sequences, where possible experimental evidence for function is described below. The GH10 endoxylanase Xyn10B that was detected at high abundance in the growth medium of cells grown in the presence of xylan [10] is expected to produce xylooligomers with arabinofuranosyl, *O*-acetyl, glucuronyl, and ferulic acid substituents [32,33,34,35]. *B. proteoclasticus* also appears to synthesize several sugar binding protein dependent, ABC-transporter systems that presumably allow it to actively transport these substituted xylooligomers across the cell membrane for further metabolism [10,13]. Such a model requires *B. proteoclasticus* to possess a range of cytosolic enzymes that are capable of processing substituted xylooligomers to their constituent monomers.

**Figure 6:**
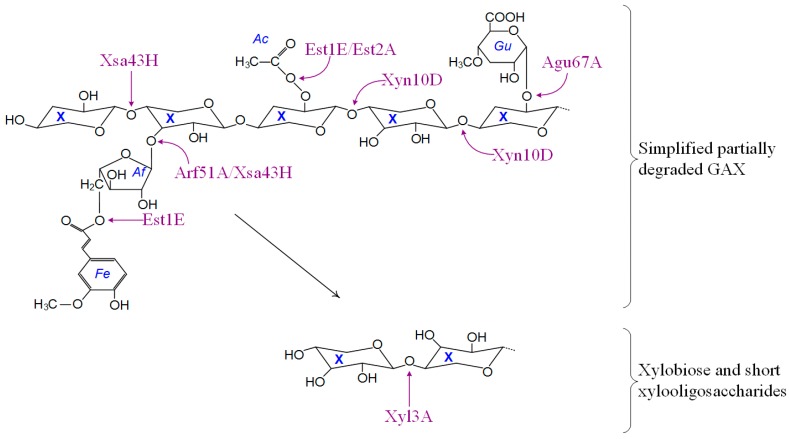
Schematic summary of the intracellular metabolism of oligosaccharides by *B. proteoclasticus*. Abbreviations: Ac, *O*-acetyl; Af, α-l-arabinofuranose; Fe, ferulic acid; GAX, glucuronoarabinoxylan; Gu, 4-*O*-methyl-d-glucuronic acid; X, xylopyranose (xylose).

The oligosaccharidases identified in the *B. proteoclasticus* cytoplasm (Table 5) include enzymes with potential to hydrolyze ferulated glucuroarabinoxylans, acetyl and glucuronic acid substituted sugars, as well as xylobiose and arabinogalactans (Figure 6), all of which are the expected products of extracellular xylan degradation [10]. Other identified oligosaccharide degrading enzymes including a glycogen phosphorylase (consistent with the demonstration of glycogen-like storage polysaccharide in B316^T^ [9]), α-amylase, pullulanase, and cellobiose phosphorylase that are implicated in the breakdown of storage polysaccharides.

**Table 5:** Predicted catalytic activities of the identified cytoplasmic oligosaccharide metabolizing enzymes.

Protein	Locus	Substrate	Reaction Catalysed ^i^
β-Galactosidase, Bga2A β-Galactosidase, Bga2B β-Galactosidase, Bga35B	Bpr_I0279 Bpr_III209 Bpr_I2006	β-d-Galactosides	Hydrolysis of terminal, non-reducing β-d-galactose residues.
β-Mannosidase, Man2A	Bpr_III237	β-d-Mannosides	Hydrolysis of terminal, non-reducing β-d-mannose residues.
β-Glucosidase, Bgl3B β-Glucosidase, Bgl3C	Bpr_I0847 Bpr_I0138	β-d-Glucosides	Hydrolysis of terminal, non-reducing β-d-glucosyl residues with release of β-d-glucose.
β-Xylosidase, Xyl3A	Bpr_I0184	1,4-β-d-Xylans	Hydrolysis of terminal, non-reducing d-xylose residues.
Cellodextrinase, Cel9B	Bpr_I1593	Cellulose, lichenin and cereal β-d-glucans	Endohydrolysis of (1→4)-β-d-glucosidic linkages.
Endo-1,4-β-xylanase and esterase, Xyn10D	Bpr_I1083	1,4-β-d-Xylans	Endohydrolysis of (1→4)-β-d-xylosidic linkages.
α-Amylase, Amy13G	Bpr_I0729	Starch and glycogen	Endohydrolysis of (1→6)-α-d-glucosidic linkages.
Glycoside hydrolase family 31, GH31C	Bpr_I1974	Unknown	Unknown.
α-Galactosidase, Aga36C	Bpr_III065	α-d-Galactosides	Hydrolysis of terminal, non-reducing α-d-galactose residues.
Xylosidase/arabinofuranosidase and esterase, Xsa43H	Bpr_I0301	α-l-Arabinosides and triacylglycerols	Hydrolysis of terminal, non-reducing D-xylose or α-l-arabinofuranoside residues/hydrolysis of triacylglycerols with release of a diacylglycerol and a carboxylate.
α-L-Arabinofuranosidase, Arf51A	Bpr_I0329	α-l-Arabinofuranosides	Hydrolysis of terminal, non-reducing α-l-arabinofuranoside residues.
α-D-Glucuronidase, Agu67A	Bpr_I0177	Glucuronoxylans	Hydrolysis of glucuronic acid substituted xylooligosaccharides.
α-L-Rhamnosidase, Rha78A	Bpr_I1686	α-l-Rhamnosides	Hydrolysis of terminal, non-reducing α-l-rhamnose residues.
Cellobiose phosphorylase, Cbp94A	Bpr_I2447	Cellobiose	Hydrolysis of cellobiose.
Feruloyl esterase, Est1E	Bpr_I2870	Esterified oligosaccharides	Deferuloylation of esterified oligosaccharides.
Acetyl-xylan esterase, Est2A	Bpr_I2939	Acetylated xylans and xylo-oligosaccharides	Deacetylation of xylans and xylo-oligosaccharides.
Glycogen phosphorylase, Glgp2	Bpr_I2847	1,4-α-d-Glucans	Exohydrolysis and phosphorylation of (1→4)-α-d-glucan.

^i^ Swiss Institute of Bioinformatics Enzyme nomenclature (http://us.expasy.org/enzyme/).

Based upon either direct functional characterization or homology to other bacterial enzymes with known catalytic activities, the diverse range of internalized substituted xylooligosaccharide are expected to be targeted by enzymes including two acetyl-xylan esterases (Est1E and Est2A), two enzymes with arabinofuranosidase catalytic potential (Arf51A and Xsa43H), at least one glucuronidase (Agu67A), and a multi-domain endoxylanase (Xyn10D). Both acetyl-xylan esterases have been functionally characterized [36,37]. Est1E is the only enzyme of its type identified in the *B. proteoclasticus* proteome and may be an important mediator of the degradation and assimilation of feruloyl xylooligosaccharides. When incubated with esterified synthetic substrates or purified rye-grass hemicellulose, Est1E released ferulic and *p*-coumaric acids [37]. In addition, a variety of ester linkages were hydrolyzed and phenolic compounds released when Est1E was incubated with birch-wood xylan, suggesting that the enzyme has wide substrate specificity. Est2A was confirmed as having true acetyl-xylan esterase activity [36], and may therefore target only acetylated xylooligosaccharides.

Homology to characterized enzymes from other bacterial species suggests that Arf51A, Agu67A, and Xsa43H are likely to mediate almost complete hydrolysis of xylooligosaccharide substituent groups. Arf51A is homologous to several *Clostridial* α-l-arabinofuranosidases [38,39,40] that preferentially hydrolyzed arabinose containing polysaccharides such as wheat arabinoxylan over other hemicellulosic substrates. Agu67A is structurally and functionally similar to several well-characterized bacterial GH67 enzymes [41,42,43] that are induced by, and are active upon, methylglucuronoxylan, which suggests that Agu67A is likely to remove 4-*O*-methylglucuronic acid residues from the backbone of assimilated methyglucuronoxylooligosaccharides. α-d-Glucuronidases cleave the α-1,2-glycosidic linkage between 4-*O*-methyl-d-glucuronic acid and the xylan backbone, and are vital for the complete degradation of hemicelluloses such as GAX. The C-terminal, GH43 domain of Xsa43H was at 65% identical to *C. stercorarium* xylosidase/arabinofuranosidase (XylA), which was particularly interesting in that at an optimal pH of 7.0 it exhibited xylosidase and arabinofuranosidase activity within the single GH43 domain [44].

Xyn10D was the only endoxylanase identified in the *B. proteoclasticus* cytosol and contains an N-terminal GH10 catalytic domain, as well a C-terminal CE10 domain, and the full-length protein is 97% identical to the *B. fibrisolvens* H17c(SA) XynB, now designated as a strain of *B. proteoclasticus* [4,45]. When expressed in *E. coli*, XynB was able to cleave the backbone of xylooligomers close to sites of arabinose substitution, which are known to negatively influence the activity of many endoxylanases upon the xylan backbone [11]. A feature of Xyn10D is that similar to Xsa43H, it contains a novel N-terminal CE10 domain implying that both enzymes might possess novel catalytic activity within the rumen microbiome.

Following the removal of xylooligomer substituents, the β-xylosidase Xyl3A that contains two non-homologous GH3 functional domains (Figure 5) is expected to complete the hydrolysis of xylobiose and xylooligosaccharides to xylose monomers that can then enter the central metabolic pathways. Xyl3A is 53% identical and 71% similar to Bxl3B from *Clostridium stercorarium* NCIMB 11754, which hydrolyzes xylose from the non-reducing end of xylotriose, liberates only small amounts of xylose from synthetic-α-arabinofuranoside, and does not release xylose from intact arabinoxylan or arabinose substituted xylooligosaccharides. Xyl3A was the only enzyme of its type identified in the *B. proteoclasticus* cytosol. Taken together these data suggest that the *B. proteoclasticus* Xyl3A may act exclusively on the non-reducing ends of short chain xylooligosaccharides and is a vital component of the cytosolic xylooligosaccharide degrading capability of *B. proteoclasticus*.

## 4. Conclusions

Proteomic examination of the *B. proteoclasticus* cytosolic proteome has provided further insight into the xylan degrading enzyme system that has evolved to enable *B. proteoclasticus* to degrade and utilize xylan [3,4], and provides a clearer understanding of the metabolic processes involved in plant cell-wall degradation by the fibrolytic rumen microbiota. Efficient xylan utilization requires the synergistic action of a variety of enzymes, including acetyl-xylan- and ferulic acid esterases, α-l-arabinofuranosidases, α-d-glucuronidases, endo-xylanases and β-xylosidases. This study confirms that *B. proteoclasticus* possesses the enzymes required for the cytosolic degradation of internalized substituted oligosaccharides that are predicted to be derived from extracellular xylan degradation [9,10,13,36,37], and supports the notion that *B. proteoclasticus* makes an important contribution to ruminant metabolism.

This study also extends our understanding of the enzymes that are likely to play important roles in xylan degradation in the rumen. The use of exogenous fibrolytic enzymes as feed additives is an emerging technology that holds promise as a means of enhancing forage utilization and improving ruminant productivity [46,47,48]. Application of fibrolytic enzymes to rumen forage prior to consumption has resulted in increased voluntary intake, milk production, and average daily weight gain as a consequence of increased forage digestibility [49,50,51,52,53,54,55]. Nonetheless, results are inconsistent and not always positive [56,57,58,59]. Commercially prepared fibrolytic enzymes for ruminant applications are cocktails of crude enzyme extracts that usually contain specified levels of xylanase or cellulase activities and are assessed primarily on their capacity to degrade plant cell walls *in vitro* [47,60], rather than their specific formulations or the suitability of these formulations for their intended purposes. In particular, it has been suggested that side-chain hydrolyzing enzymes, such as ferulic acid esterases, acetyl-xylan esterases and arabinofuranosidases, should be incorporated [47]. This study has provided a clearer understanding of the specific enzymes that are utilized by a prominent hemicellulose degrading rumen microbe, and may assist in achieving the more rational and targeted design of multi-enzyme products aimed at enhancing the cell wall degradation of rumen forages.

## Supplementary Materials

Supplementary File 1
