# Supplementary material for: The Cytosolic Oligosaccharide-Degrading Proteome of Butyrivibrio Proteoclasticus
Source: Proteomes. 2015 Oct 27;3(4):347–68. doi: 10.3390/proteomes3040347 (PMC5217386; doi:10.3390/proteomes3040347)
Supplement: Supplementary File 1 [file proteomes-03-00347-s001.pdf]

Supplementary Materials

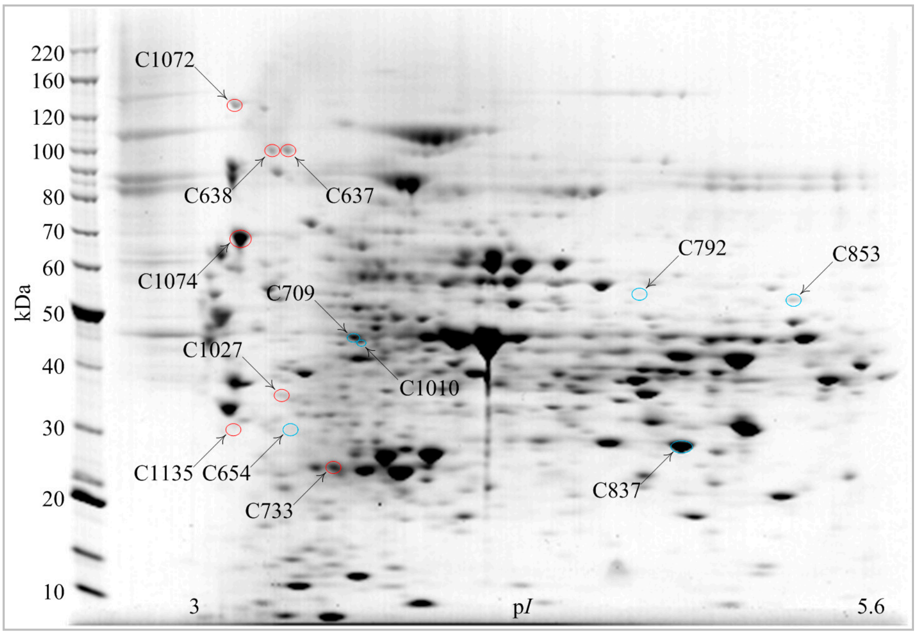

Figure S1. Differentially abundant spots between the mid-log phase cells grown on xylan or xylose. Red and blue circles denote spots with increased and decreased abundance respectively in the xylan growth condition. The identities of the circled spots are summarized in Table 3.

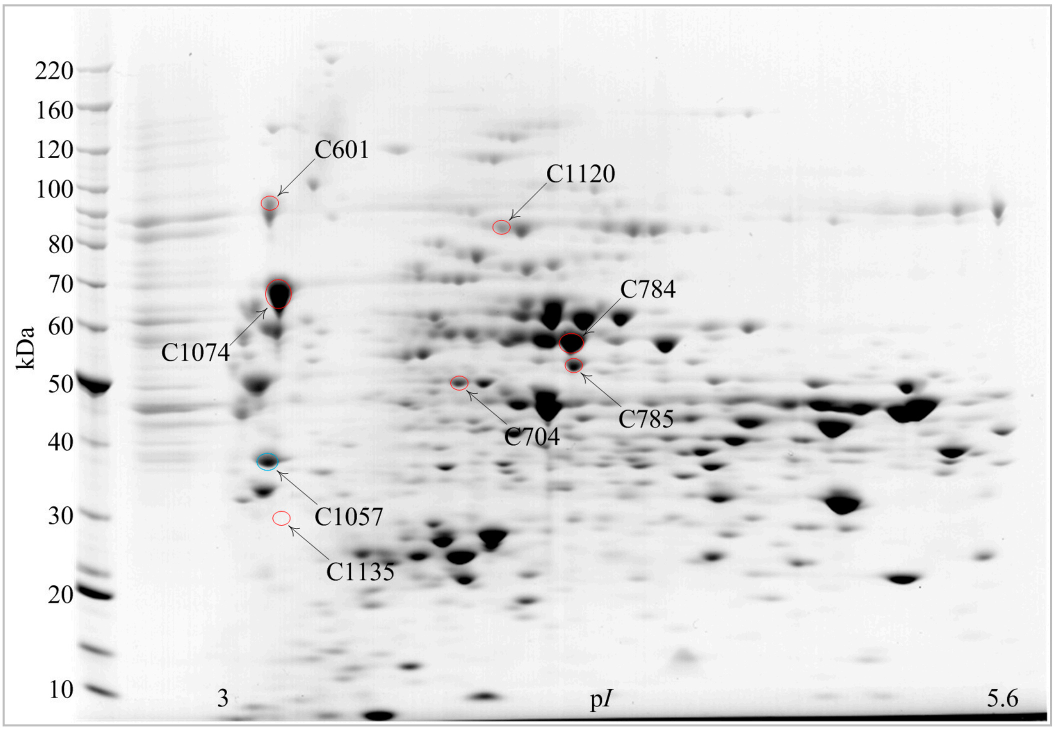

Figure S2. Differentially abundant spots between the stationary phase cells grown on xylan or xylose. Red and blue circles denote spots with increased and decreased abundance respectively in the xylan growth condition. The identities of the circled spots are summarized in Table 4.

Table S1. Summary of the non-polysaccharide degrading proteins identified in the *B. proteoclasticus* cytosol by 2DE/MALDI-TOF.

Protein	Locus	Location	Score	p*I*	kDa	Pep.	Cov.
**Amino Acid Biosynthesis**							
Acetylornithine aminotransferase, ArgD	Bpr_I1809	C	1.7×10^{-4}	5.1	43.9	11	34%
Aspartate/tyrosine/aromatic aminotransferase	Bpr_I2631	C	3.0×10^{-14}	4.7	43.8	15	46%
Aspartate-semialdehyde dehydrogenase, Asd	Bpr_I1664	C	7.6×10^{-18}	5.5	40.1	17	50%
Branched-chain amino acid aminotransferase, IlvE	Bpr_I1650	C	2.4×10^{-12}	5.2	39.2	13	32%
Cysteine synthase, CysK	Bpr_I1089	C	1.9×10^{-13}	5.0	32.3	18	72%
Diaminopimelate dehydrogenase	Bpr_I0298	C	9.6×10^{-16}	5.6	35.8	16	49%
Dihydrodipicolinate reductase, DapB	Bpr_I2453	C	2.7×10^{-6}	4.9	27.0	9	46%
Glu/Leu/Phe/Val dehydrogenase	Bpr_I2129	C	1.2×10^{-30}	5.4	48.6	31	64%
Imidazole glycerol phosphate synthase glutamine amidotransferase subunit	Bpr_I1240	C	8.0×10^{-3}	4.7	22.5	8	44%
Ketol-acid reductoisomerase, IlvC	Bpr_I1657	C	3.8×10^{-16}	5.1	37.3	20	64%
NADPH-dependent glutamate synthase, GltA3	Bpr_I1306	C	1.9×10^{-4}	5.0	49.3	8	20%
OAH/OAS sulfhydrylase	Bpr_I2467	C	9.6×10^{-19}	5.6	45.9	16	56%
Phosphoserine aminotransferase, SerC	Bpr_I1341	C	2.4×10^{-15}	5.0	40.1	20	59%
Pyrroline-5-carboxylate reductase, ProC	Bpr_I2765	C	4.6×10^{-4}	5.2	28.1	7	29%
Threonine synthase, ThrC	Bpr_I1058	C	1.5×10^{-10}	4.8	54.4	17	48%
**Carbohydrate Metabolism**							
1-phosphofructokinase, PfkB	Bpr_I2103	C	4.8×10^{-13}	5.0	32.4	13	56%
2,3-bisphosphoglycerate-independent phosphoglycerate mutase, GpmA	Bpr_I1294	C	6.1×10^{-18}	4.7	56.5	18	48%
2-dehydro-3-deoxygluconokinase, KdgK	Bpr_I0932	C	3.8×10^{-14}	5.5	37.2	18	34%
2-keto-3-deoxygluconate 6-phosphate aldolase/2-keto-4-hydroxyglutarate aldolase	Bpr_I0931	C	3.0×10^{-14}	5.7	34.3	17	62%
5-keto 4-deoxyuronate isomerase, KduI	Bpr_I0929	C	8.5×10^{-5}	4.8	32.7	13	45%
6-phosphofructokinase, PfkA1	Bpr_I0224	C	1.5×10^{-8}	7.1	39.0	14	37%
6-phosphofructokinase, PfkA4	Bpr_I2767	C	4.0×10^{-7}	8.1	34.8	14	41%
6-phosphogluconolactonase	Bpr_I1331	C	6.3×10^{-5}	4.8	39.3	8	20%
Aldose 1-epimerase	Bpr_I0228	C	3.0×10^{-15}	4.8	38.3	16	56%
Aldose 1-epimerase family protein	Bpr_I1782	C	7.6×10^{-13}	5.3	33.9	14	46%
Altronate oxidoreductase, UxaB	Bpr_I1275	C	$2.4 \times 10^{-1\circ}$	4.6	56.0	16	36%
Deoxyribose-phosphate aldolase, DeoC	Bpr_I1062	C	7.6×10^{-16}	5.2	24.0	15	77%
Fructose-1,6-bisphosphate aldolase, FbaA	Bpr_I2903	C	3.0×10^{-13}	5.1	30.5	14	49%
Galactokinase, GalK	Bpr_I2843	C	1.4×10^{-3}	4.6	43.4	7	16%
Glucose-6-phosphate isomerase, Gpi	Bpr_I0035	C	1.2×10^{-15}	5.0	57.1	16	41%
Glyceraldehyde-3-phosphate dehydrogenase, Gap	Bpr_I2050	C	7.6×10^{-10}	5.7	36.9	11	34%
Lactaldehyde reductase, FucO	Bpr_I2065	C	1.8×10^{-3}	4.7	41.2	9	17%
L-fucose isomerase related protein	Bpr_I0185	C	1.5×10^{-11}	4.9	55.4	17	39%
Phosphoenolpyruvate carboxykinase, PckA	Bpr_I0091	C	1.5×10^{-14}	4.9	59.5	18	32%
Phosphoglycerate kinase, Pgk	Bpr_I2049	C	6.1×10^{-19}	5.4	43.7	22	59%
Pyruvate kinase, PykA	Bpr_I0083	C	1.3×10^{-3}	4.8	51.7	9	20%
Transaldolase	Bpr_I1511	C	5.9×10^{-7}	4.8	23.4	11	71%

Table S1. *Cont.*

Protein	Locus	Location	Score	p*I*	kDa	Pep.	Cov.
**Carbohydrate Metabolism**							
Transketolase subunit A, TktA3	Bpr_I2813	C	3.4×10^{-2}	5.4	30.0	6	19%
Transketolase subunit B, TktB3	Bpr_I2812	C	2.4×10^{-7}	5.5	33.0	10	37%
Triosephosphate isomerase, TpiA	Bpr_I2048	C	1.2×10^{-18}	4.6	26.8	18	64%
UDP-galactose 4-epimerase, GalE	Bpr_I0192	C	4.8×10^{-14}	5.2	37.2	18	47%
UTP-glucose-1-phosphate uridylyltransferase, GalU	Bpr_I0191	C	1.9×10^{-20}	4.6	45.8	23	56%
Xylulokinase, XylB	Bpr_I0173	C	1.5×10^{-10}	4.9	53.7	14	38%
**Cell Cycle**							
DNA polymerase III β-subunit, DnaN	Bpr_I0002	C	1.2×10^{-13}	4.7	41.4	17	50%
Single-stranded DNA binding protein, Ssb1	Bpr_I0240	C	7.6×10^{-10}	4.7	16.6	12	63%
**Cell Envelope**							
Aminotransferase DegT/DnrJ/EryC1/StrS family	Bpr_I0345	C	4.8×10^{-11}	5.8	44.9	14	33%
Aminotransferase DegT/DnrJ/EryC1/StrS family	Bpr_I2311	C	3.8×10^{-10}	5.0	51.0	18	35%
Aminotransferase DegT/DnrJ/EryC1/StrS family	Bpr_I2543	C	1.2×10^{-8}	5.1	47.3	14	37%
Cell wall binding domain-containing protein	Bpr_I0264	S	3.0×10^{-16}	4.3	195.3	31	19%
dTDP-4-dehydrorhamnose 3,5-epimerase, RfbC2	Bpr_I0548	C	9.6×10^{-11}	4.7	20.6	11	49%
dTDP-4-dehydrorhamnose reductase, RfbD1	Bpr_I2582	C	3.0×10^{-7}	4.8	32.1	9	41%
Glycosyl transferase GT2 family	Bpr_I2565	C	2.3×10^{-2}	5.2	38.1	8	29%
NAD dependent epimerase/dehydratase	Bpr_I2647	C	1.5×10^{-14}	5.5	38.8	17	48%
NAD dependent epimerase/dehydratase	Bpr_I2930	C	4.8×10^{-11}	8.8	31.5	11	44%
NAD-dependent epimerase/dehydratase	Bpr_I0517	C	1.9×10^{-21}	4.9	42.0	18	51%
NAD-dependent epimerase/dehydratase	Bpr_I2310	C	1.5×10^{-2}	5.0	34.2	10	33%
NAD-dependent epimerase/dehydratase	Bpr_I2537	C	1.5×10^{-13}	4.7	36.0	14	52%
Nucleotide sugar dehydrogenase	Bpr_I0828	C	4.7×10^{-2}	5.0	46.1	8	18%
Phosphoglucomutase/phosphomannomutase family protein	Bpr_I0554	C	7.6×10^{-9}	4.7	66.0	15	20%
S-adenosyl-methyltransferase, MraW	Bpr_I1869	C	1.9×10^{-9}	7.0	35.3	13	41%
**Cellular Processes**							
Chemotaxis protein methyltransferase, CheR	Bpr_I2033	C	1.5×10^{-9}	8.4	30.5	14	42%
Chemotaxis-specific methylesterase, CheB	Bpr_I1384	C	4.6×10^{-6}	8.1	38.6	10	32%
Flagellar hook protein, FlgE1	Bpr_I1367	C	1.5×10^{-4}	4.2	112.3	12	14%
Flagellar motor switch protein, FliG	Bpr_I1359	C	9.6×10^{-9}	4.3	38.6	9	33%
Superoxide dismutase, SodA	Bpr_I0467	C	1.9×10^{-13}	5.3	24.1	11	61%
**Central Metabolism**							
Agmatine deiminase, AguA	Bpr_I1200	C	1.2×10^{-8}	4.2	49.5	12	30%
Carboxynorspermidine dehydrogenase	Bpr_I1198	C	6.9×10^{-2}	4.6	47.4	9	20%
N-carbamoylputrescine amidohydrolase, AguB	Bpr_I1201	C	4.8×10^{-15}	5.0	33.8	16	56%
S-adenosylmethionine synthetase, MetK	Bpr_I2769	C	4.8×10^{-16}	4.9	43.1	16	38%
**Energy Metabolism**							
2-enoate reductase	Bpr_I1763	C	1.3×10^{-4}	7.3	76.0	9	17%
2-enoate reductase	Bpr_I1977	C	4.8×10^{-2}	6.2	77.4	4	12%
3-hydroxybutyryl-CoA dehydrogenase, Hbd	Bpr_I2486	S	6.1×10^{-14}	5.2	31.2	13	47%
ATP synthase F1 α-subunit, AtpA1	Bpr_I0166	C	$1.9 \times 10^{-1\circ}$	5.3	55.0	15	31%

Table S1. *Cont.*

Protein	Locus	Location	Score	p*I*	kDa	Pep.	Cov.
**Energy Metabolism**							
ATP synthase F1 β-subunit, AtpD1	Bpr_I0168	C	4.8×10^{-16}	4.6	50.3	15	41%
Butyrate kinase, Buk	Bpr_I2323	C	6.1×10^{-22}	5.1	38.7	18	66%
Butyryl-CoA dehydrogenase, Bcd	Bpr_I2485	C	3.8×10^{-17}	6.1	42.5	21	60%
Crotonase, Crt	Bpr_I2487	C	1.2×10^{-14}	4.7	28.0	19	65%
Electron transfer flavoprotein α-subunit, EtfA	Bpr_I2483	C	1.2×10^{-8}	4.8	37.4	12	36%
Electron transfer flavoprotein β-subunit, EtfB	Bpr_I2484	C	1.5×10^{-11}	5.2	28.4	14	53%
Isocitrate dehydrogenase, Icd	Bpr_I1102	C	4.8×10^{-13}	5.1	45.1	18	42%
Methylmalonyl-CoA decarboxylase α-subunit, MmdA	Bpr_I1226	C	3.9×10^{-7}	4.5	50.9	12	28%
Phosphate acetyltransferase, Pta	Bpr_III011	C	1.3×10^{-2}	5.2	14.4	7	54%
Phosphate butyryltransferase, Ptb	Bpr_I2324	C	4.8×10^{-16}	5.1	33.3	19	72%
Pyruvate formate lyase, PflB	Bpr_I0112	C	3.0×10^{-24}	5.7	84.9	30	50%
Pyruvate phosphate dikinase, PpdK	Bpr_I1154	C	7.6×10^{-15}	4.8	104.3	28	28%
Pyruvate:ferredoxin oxidoreductase	Bpr_I0269	C	2.4×10^{-14}	5.2	127.3	25	21%
Thiolase, ThlA1	Bpr_I2488	C	2.4×10^{-14}	5.0	42.0	15	51%
Thiolase, ThlA2	Bpr_I2475	S	3.8×10^{-8}	6.0	41.0	12	24%
Thioredoxin-disulfide reductase, TrxB	Bpr_I2491	C	9.6×10^{-8}	4.5	33.7	13	43%
**Hypothetical**							
Hypothetical protein	Bpr_I0280	C	3.8×10^{-15}	8.4	23.2	16	76%
Hypothetical protein	Bpr_I0417	C	7.1×10^{-3}	4.9	39.8	11	29%
Hypothetical protein	Bpr_I0592	C	$6.1 \times 10^{-1\circ}$	7.3	32.3	11	38%
Hypothetical protein	Bpr_I0874	C	1.0×10^{-5}	4.5	14.8	5	36%
Hypothetical protein	Bpr_I0962	C	3.8×10^{-9}	4.3	17.3	9	49%
Hypothetical protein	Bpr_I1177	C	2.4×10^{-11}	4.3	24.7	12	65%
Hypothetical protein	Bpr_I1179	C	1.2×10^{-7}	4.7	24.9	11	45%
Hypothetical protein	Bpr_I1180	C	9.6×10^{-18}	7.4	36.4	18	53%
Hypothetical protein	Bpr_I1213	C	1.4×10^{-5}	4.3	26.5	8	39%
Hypothetical protein	Bpr_I1281	C	1.5×10^{-13}	4.6	34.1	18	52%
Hypothetical protein	Bpr_I1477	C	2.9×10^{-4}	4.2	19.1	5	49%
Hypothetical protein	Bpr_I1608	C	3.1×10^{-6}	4.6	47.5	10	26%
Hypothetical protein	Bpr_I2291	S	7.1×10^{-7}	4.2	42.6	8	25%
Hypothetical protein	Bpr_I2455	C	6.1×10^{-26}	4.7	47.7	26	58%
Hypothetical protein	Bpr_I2494	C	6.1×10^{-8}	4.3	33.0	11	41%
Hypothetical protein	Bpr_I2583	S	8.7×10^{-6}	4.3	44.3	9	24%
Hypothetical protein	Bpr_I2606	C	4.2×10^{-5}	4.8	41.1	13	31%
Hypothetical protein	Bpr_I2608	C	6.1×10^{-16}	7.3	32.8	17	44%
Hypothetical protein	Bpr_I2619	C	3.8×10^{-8}	4.5	8.6	6	46%
Hypothetical protein	Bpr_I2634	C	4.8×10^{-7}	9.8	16.4	8	50%
Hypothetical protein	Bpr_I2744	C	2.4×10^{-8}	4.8	28.5	10	51%
Hypothetical protein	Bpr_III133	C	1.6×10^{-3}	5.6	57.9	10	16%
**Lipid Metabolism**							
3-oxoacyl-(acyl-carrier-protein) synthase, FabF	Bpr_I1268	C	2.0×10^{-6}	5.3	44.5	11	28%
Glycerol kinase, GlpK	Bpr_I1744	C	3.6×10^{-5}	4.9	55.6	9	18%

Table S1. *Cont.*

Protein	Locus	Location	Score	p*I*	kDa	Pep.	Cov.
**Nucleic Acid Metabolism**							
Excinuclease ABC A subunit, UvrA	Bpr_I2597	C	3.8×10^{-12}	7.4	106.8	20	26%
Type I restriction modification system S subunit	Bpr_IV100	C	1.5×10^{-2}	6.1	46.5	7	18%
**Nucleotide Metabolism**							
5-aminoimidazole-4-carboxamide ribonucleotide transformylase	Bpr_I0732	C	1.5×10^{-12}	5.1	44.4	12	38%
Adenylate kinase, Adk	Bpr_I0615	C	1.5×10^{-10}	5.1	23.8	12	72%
Adenylosuccinate lyase, PurB	Bpr_I2212	C	1.2×10^{-10}	5.4	53.7	16	28%
Dihydroorotate dehydrogenase, PyrD	Bpr_I2788	C	7.6×10^{-10}	5.1	32.3	13	47%
IMP cyclohydrolase, PurO	Bpr_I0731	C	4.0×10^{-6}	4.7	32.2	7	21%
Inosine-uridine preferring nucleoside hydrolase	Bpr_I0724	C	7.4×10^{-3}	4.5	32.9	9	25%
Phosphoribosylamine-glycine ligase, PurD	Bpr_I0870	C	1.8×10^{-6}	4.6	46.3	12	29%
Phosphoribosylaminoimidazole-succinocarboxamide synthase, PurC	Bpr_I1144	C	3.8×10^{-18}	4.7	33.3	21	75%
Purine nucleoside phosphorylase, DeoD2	Bpr_I2948	C	4.8×10^{-13}	5.1	26.1	16	55%
Purine-nucleoside phosphorylase, DeoD1	Bpr_I1527	C	3.0×10^{-12}	4.6	29.5	13	67%
Uridine phosphorylase, Udp	Bpr_I1561	C	9.6×10^{-13}	5.4	28.1	14	69%
Uridylate kinase, PyrH	Bpr_I0953	C	1.9×10^{-13}	5.4	25.5	15	55%
**Plasmid Functions**							
Single stranded DNA binding protein, Ssb3	Bpr_II423	C	1.9×10^{-10}	4.7	16.3	12	60%
**Protein Fate**							
Chaperone protein, DnaK	Bpr_I2625	C	9.6×10^{-10}	4.5	66.8	16	28%
Chaperonin, GroEL	Bpr_I1193	C	1.1×10^{-6}	5.0	60.0	19	32%
Oligoendopeptidase, PepF1	Bpr_I0779	C	1.9×10^{-13}	4.7	68.9	22	32%
Peptidase M16 family	Bpr_I1215	C	3.0×10^{-39}	4.6	111.5	44	46%
Peptidase U62 family	Bpr_I2456	C	6.1×10^{-9}	4.8	52.3	15	33%
Peptidyl-prolyl *cis*-trans isomerase FKBP-type	Bpr_I1031	C	2.5×10^{-6}	4.1	33.4	8	25%
Serine protease subtilisin family	Bpr_I2629	S	2.8×10^{-5}	3.8	153.3	15	12%
**Protein Synthesis**							
Aspartyl-tRNA synthetase, AspS	Bpr_I0140	C	3.8×10^{-14}	4.7	69.2	23	35%
Endoribonuclease L-PSP	Bpr_I2149	C	2.8×10^{-5}	4.4	13.5	4	56%
Methionyl-tRNA synthetase, MetG	Bpr_I2807	C	9.6×10^{-9}	4.9	76.4	21	32%
Polyribonucleotide nucleotidyltransferase	Bpr_I0975	C	7.6×10^{-15}	4.9	76.5	21	37%
Ribosomal protein L1, RplA	Bpr_I2299	C	1.2×10^{-12}	9.8	24.6	16	58%
Ribosomal protein L10, RplJ	Bpr_I2371	C	1.8×10^{-4}	4.6	20.4	8	32%
Ribosomal protein L13, RplM	Bpr_I0630	C	6.1×10^{-8}	9.9	16.2	11	77%
Ribosomal protein L18, RplR	Bpr_I0610	C	3.0×10^{-6}	10.5	13.5	9	48%
Ribosomal protein L21, RplU	Bpr_I1443	C	7.6×10^{-3}	10.3	11.3	5	41%
Ribosomal protein L22, RplV	Bpr_I0599	C	4.4×10^{-6}	10.7	15.1	8	56%
Ribosomal protein L3, RplC	Bpr_I0594	C	1.9×10^{-24}	10.6	24.3	25	84%
Ribosomal protein L4, RplD	Bpr_I0595	C	4.0×10^{-7}	10.2	22.9	9	47%
Ribosomal protein L5, RplE	Bpr_I0606	C	3.8×10^{-11}	9.8	20.4	15	63%
Ribosomal protein L6, RplF	Bpr_I0609	C	2.4×10^{-15}	10.3	19.4	14	60%

Table S1. *Cont.*

Protein	Locus	Location	Score	p*I*	kDa	Pep.	Cov.
**Protein Synthesis**							
Ribosomal protein S1, RpsA	Bpr_I2035	C	1.5×10^{-19}	4.6	41.9	21	54%
Ribosomal protein S2, RpsB	Bpr_I1395	C	9.6×10^{-9}	8.4	27.6	11	39%
Ribosomal protein S3, RpsC	Bpr_I0600	C	9.6×10^{-8}	9.6	24.6	12	53%
Ribosomal protein S4, RpsD	Bpr_I0622	C	2.4×10^{-11}	10.6	24.1	13	43%
Ribosomal protein S5, RpsE	Bpr_I0611	C	3.0×10^{-17}	10.5	17.9	15	79%
Ribosomal protein S7, RpsG	Bpr_I2366	C	1.2×10^{-11}	10.5	17.5	13	70%
Ribosomal protein S9, RpsI	Bpr_I0631	C	3.8×10^{-13}	11.1	15.4	11	67%
Single stranded nucleic acid binding protein	Bpr_I2957	C	3.0×10^{-10}	6.3	28.3	14	52%
Threonyl-tRNA synthetase, ThrZ	Bpr_I2374	C	1.2×10^{-11}	5.0	73.7	22	26%
Translation elongation factor EF-G-like protein	Bpr_I1098	C	3.0×10^{-26}	4.9	77.3	31	46%
Translation elongation factor G, FusA	Bpr_I2365	C	9.6×10^{-15}	4.6	78.0	21	27%
Translation elongation factor Ts, Tsf	Bpr_I1396	C	3.0×10^{-3}	4.8	34.9	10	33%
Translation elongation factor Tu, TufA	Bpr_I2364	C	3.0×10^{-21}	4.8	43.6	19	62%
tRNA (guanine-N7)-methyltransferase, TrmB	Bpr_I2923	C	4.4×10^{-7}	7.6	25.1	10	51%
**Regulation**							
Anti-sigma factor antagonist	Bpr_I0800	C	4.9×10^{-7}	4.2	10.8	7	89%
Anti-sigma factor antagonist/phosphotransferase domain-containing protein	Bpr_I0249	C	9.6×10^{-14}	4.6	49.8	20	48%
Cold shock domain protein, CspD1	Bpr_I2451	C	2.3×10^{-6}	4.6	7.3	5	89%
**Signal transduction**							
GGDEF domain-containing protein	Bpr_I1183	C	1.8×10^{-5}	4.6	53.0	10	24%
Hpr kinase/phosphatase, HprK	Bpr_I0099	C	1.3×10^{-4}	5.1	35.9	11	40%
PhoH family protein	Bpr_I1309	C	1.2×10^{-7}	8.6	38.6	10	34%
S-ribosylhomocysteinase, LuxS	Bpr_I0033	C	7.6×10^{-13}	5.0	18.2	11	54%
**Transcription**							
DNA directed RNA polymerase β-subunit, RpoB	Bpr_I2369	C	9.6×10^{-13}	4.6	145.5	22	20%
DNA directed RNA polymerase β'-subunit, RpoC	Bpr_I2368	C	1.5×10^{-23}	6.6	139.3	36	30%
DNA-directed RNA polymerase α-subunit, RpoA	Bpr_I0623	C	3.8×10^{-18}	4.4	35.1	17	58%
Transcription antitermination factor, NusB	Bpr_I0979	C	7.3×10^{-7}	4.3	15.3	8	57%
**Transporters**							
ABC transporter ATP-binding protein	Bpr_I2802	C	6.1×10^{-11}	6.7	41.1	17	43%
Amino acid ABC transporter substrate-binding protein	Bpr_I1826	S	5.8×10^{-4}	4.0	31.6	7	21%
Oligopeptide ABC transporter substrate-binding protein, OppA1	Bpr_I1276	S	1.2×10^{-20}	4.0	83.1	25	35%
Peptide/nickel ABC transporter periplasmic protein	Bpr_I2750	S	8.0×10^{-7}	3.9	57.9	9	23%
Peptide/nickel ABC transporter substrate-binding protein	Bpr_I1860	S	7.6×10^{-11}	4.1	58.4	16	41%
Sugar ABC transporter substrate-binding protein	Bpr_I0182	S	7.6×10^{-7}	4.1	63.3	13	27%
Sugar ABC transporter substrate-binding protein	Bpr_I0237	S	8.5×10^{-3}	3.9	64.8	6	14%
Sugar ABC transporter substrate-binding protein	Bpr_I0313	S	1.1×10^{-3}	3.8	55.2	7	11%
Sugar ABC transporter substrate-binding protein	Bpr_I0937	S	9.6×10^{-14}	4.1	61.3	15	35%
Sugar ABC transporter substrate-binding protein	Bpr_I1589	S	7.3×10^{-6}	3.9	61.1	13	34%
Sugar ABC transporter substrate-binding protein	Bpr_I1667	S	7.6×10^{-11}	3.9	47.8	11	42%

Table S1. *Cont.*

Protein	Locus	Location	Score	p*I*	kDa	Pep.	Cov.
**Transporters**							
Sugar ABC transporter substrate-binding protein	Bpr_I1720	S	7.4×10^{-4}	4.0	49.3	9	18%
Sugar ABC transporter substrate-binding protein	Bpr_I2010	S	1.9×10^{-6}	3.7	47.3	9	35%
Sugar ABC transporter substrate binding protein	Bpr_I2344	S	4.4×10^{-4}	3.9	52.3	10	27%
Sugar ABC transporter substrate-binding protein	Bpr_I2443	S	1.9×10^{-11}	4.0	57.0	16	31%
Xylose ABC transporter substrate-binding protein	Bpr_I1173	S	9.6×10^{-12}	4.2	38.4	14	57%
**Unknown function**							
Acetyltransferase	Bpr_I1661	C	4.8×10^{-8}	6.7	24.9	10	49%
Acetyltransferase GNAT family	Bpr_I0656	C	3.0×10^{-7}	6.7	21.0	9	51%
Aminotransferase domain-containing protein	Bpr_I1980	C	1.5×10^{-11}	5.5	48.3	12	24%
FAD dependent oxidoreductase	Bpr_I2038	C	2.4×10^{-6}	6.7	45.2	10	26%
Hydrolase α/β-fold family	Bpr_I2806	C	6.1×10^{-8}	8.6	36.5	14	46%
NUDIX domain-containing protein	Bpr_I1938	C	7.6×10^{-12}	4.2	18.8	12	70%
Phosphoribulokinase/uridine kinase family protein	Bpr_I0923	C	1.9×10^{-16}	6.9	63.1	18	35%
Pyridoxamine 5′-phosphate oxidase family protein	Bpr_I1023	C	1.2×10^{-5}	5.3	15.0	6	61%
UBA/TS-N domain-containing protein	Bpr_III040	C	4.5×10^{-7}	4.5	13.6	8	42%
**Vitamins and Cofactors**							
Cobalamin biosynthesis protein. CobW1	Bpr_I2021	C	3.8×10^{-8}	4.4	36.9	9	32%
FeS assembly protein. SufD	Bpr_I0053	C	7.6×10^{-12}	4.5	42.7	11	37%
Methylenetetrahydrofolate dehydrogenase/ cyclohydrolase. FolD	Bpr_I1303	C	1.5×10^{-5}	5.0	30.6	9	27%

Table S2. Summary of the non-polysaccharidase degrading proteins identified in the *B. proteoclasticus* cytosol by LC-MS/MS.

Protein	Locus	Location	Score	p*I*	kDa	Cov.
**Amino Acid Biosynthesis**						
Anthranilate phosphoribosyltransferase, TrpD	Bpr_I0012	C	1.5×10^{-12}	5.1	36.6	26%
Aspartate/tyrosine/aromatic aminotransferase	Bpr_I2631	C	2.3×10^{-13}	4.7	43.8	18%
Aspartate-semialdehyde dehydrogenase, Asd	Bpr_I1664	C	8.2×10^{-14}	5.5	40.1	54%
ATP phosphoribosyltransferase regulatory subunit, HisZ	Bpr_I1451	C	2.0×10^{-6}	4.5	41.3	11%
Branched-chain amino acid aminotransferase, IlvE	Bpr_I1650	C	3.4×10^{-8}	5.2	39.2	21%
Chorismate mutase/prephenate dehydratase, PheA	Bpr_I1730	C	1.4×10^{-9}	5.0	42.4	13%
Cysteine synthase, CysK	Bpr_I1089	C	8.9×10^{-9}	5.0	32.3	14%
D-3-phosphoglycerate dehydrogenase, SerA	Bpr_I1342	C	4.4×10^{-13}	5.3	42.0	26%
Diaminopimelate decarboxylase, LysA	Bpr_I1243	C	4.8×10^{-12}	4.8	48.0	9%
Diaminopimelate dehydrogenase	Bpr_I0298	C	9.9×10^{-15}	5.6	35.8	41%
Diaminopimelate epimerase, DapF	Bpr_I2388	C	2.2×10^{-5}	4.4	33.5	6%
Dihydroxy-acid dehydratase, IlvD	Bpr_I1338	C	$1.0 \times 10^{-3\circ}$	6.0	58.9	30%
Glu/Leu/Phe/Val dehydrogenase	Bpr_I2129	C	$1.0 \times 10^{-3\circ}$	5.4	48.6	57%
Histidinol dehydrogenase, HisD	Bpr_I1449	C	7.8×10^{-9}	4.8	47.0	12%
Ketol-acid reductoisomerase, IlvC	Bpr_I1657	C	2.2×10^{-15}	5.1	37.3	64%
NADPH-dependent glutamate synthase, GltA3	Bpr_I1306	C	1.0×10^{-30}	5.0	49.3	17%
OAH/OAS sulfhydrylase	Bpr_I0317	C	6.4×10^{-11}	5.4	45.6	12%

Table S2. *Cont.*

Protein	Locus	Location	Score	p*I*	kDa	Cov.
**Amino Acid Biosynthesis**						
OAH/OAS sulfhydrylase	Bpr_I2467	C	2.1×10^{-14}	5.6	45.9	25%
Phosphoserine aminotransferase, SerC	Bpr_I1341	C	3.8×10^{-14}	5.0	40.1	51%
Phosphoserine phosphatase/homoserine phosphotransferase bifunctional protein, ThrH	Bpr_I1141	C	1.4×10^{-11}	4.5	22.4	10%
Serine hydroxymethyltransferase, GlyA	Bpr_I1796	C	8.6×10^{-13}	6.2	44.9	21%
Threonine dehydratase, IlvA	Bpr_I1571	C	5.4×10^{-10}	6.0	44.4	8%
Threonine synthase, ThrC	Bpr_I1058	C	1.1×10^{-16}	4.8	54.4	35%
Tryptophan synthase α-subunit, TrpA	Bpr_I0008	C	2.0×10^{-7}	4.6	27.7	10%
Tryptophan synthase β-subunit, TrpB2	Bpr_I1140	C	1.1×10^{-8}	5.4	50.6	11%
**Carbohydrate Metabolism**						
1-phosphofructokinase, PfkB	Bpr_I2103	C	4.1×10^{-12}	5.0	32.4	14%
2,3-bisphosphoglycerate-independent phosphoglycerate mutase, GpmA	Bpr_I1294	C	1.0×10^{-30}	4.7	56.5	51%
2-dehydro-3-deoxygluconokinase, KdgK	Bpr_I0932	C	1.4×10^{-7}	5.5	37.2	4%
2-deoxy-D-gluconate 3-dehydrogenase, KduD	Bpr_I0930	C	1.6×10^{-8}	4.6	30.2	16%
2-keto-3-deoxygluconate 6-phosphate aldolase/2-keto-4-hydroxyglutarate aldolase	Bpr_I0931	C	1.5×10^{-12}	5.7	34.3	18%
6-phosphofructokinase, PfkA2	Bpr_I1068	C	5.6×10^{-14}	5.4	46.0	28%
6-phosphofructokinase, PfkA4	Bpr_I2767	C	3.3×10^{-14}	8.1	34.8	23%
6-phosphogluconolactonase	Bpr_I1331	C	1.4×10^{-12}	4.8	39.3	14%
Aldose 1-epimerase	Bpr_I0228	C	1.5×10^{-13}	4.8	38.3	32%
Altronate oxidoreductase, UxaB	Bpr_I1275	C	3.8×10^{-11}	4.6	56.0	7%
Deoxyribose-phosphate aldolase, DeoC	Bpr_I1062	C	8.6×10^{-14}	5.2	24.0	43%
Fructose-1,6-bisphosphate aldolase FbaA	Bpr_I2903	C	1.0×10^{-30}	5.1	30.5	77%
Glucokinase, Glk	Bpr_I0100	C	2.5×10^{-10}	4.9	32.9	19%
Glucose-1-phosphate adenylyltransferase, GlgC1	Bpr_I0290	C	8.1×10^{-10}	4.7	46.9	18%
Glucose-6-phosphate isomerase, Gpi	Bpr_I0035	C	1.0×10^{-30}	5.0	57.1	53%
Glucuronate isomerase, UxaC	Bpr_I1591	C	1.9×10^{-12}	5.0	54.4	13%
Glyceraldehyde-3-phosphate dehydrogenase, Gap	Bpr_I2050	C	1.0×10^{-30}	5.7	36.9	41%
Glycogen synthase ADP-glucose type, GlgA	Bpr_I1257	C	1.3×10^{-7}	5.6	56.0	9%
Lactaldehyde reductase, FucO	Bpr_I2065	C	1.1×10^{-15}	4.7	41.2	35%
L-fucose isomerase related protein	Bpr_I0185	C	1.0×10^{-30}	4.9	55.4	62%
L-ribulose-5-phosphate 4-epimerase, AraD	Bpr_I2814	C	1.0×10^{-10}	6.0	26.0	11%
Phosphoenolpyruvate carboxykinase, PckA	Bpr_I0091	C	1.0×10^{-30}	4.9	59.5	56%
Phosphoglycerate kinase, Pgk	Bpr_I2049	C	1.0×10^{-30}	5.4	43.7	69%
Pyruvate kinase, PykA	Bpr_I0083	C	1.6×10^{-8}	4.8	51.7	9%
Ribose-phosphate pyrophosphokinase, PrsA1	Bpr_I0286	C	2.7×10^{-13}	5.7	45.7	22%
Transaldolase	Bpr_I1511	C	4.7×10^{-14}	4.8	23.4	70%
Transketolase subunit A, TktA3	Bpr_I2813	C	1.1×10^{-16}	5.4	30.0	29%
Transketolase subunit B, TktB3	Bpr_I2812	C	1.0×10^{-30}	5.5	33.0	37%
Triosephosphate isomerase, TpiA	Bpr_I2048	C	1.0×10^{-30}	4.6	26.8	84%
Xylulokinase, XylB	Bpr_I0173	C	1.8×10^{-14}	4.9	53.7	27%

Table S2. *Cont.*

Protein	Locus	Location	Score	p*I*	kDa	Cov.
**Cell Cycle**						
Cell division protein, FtsZ	Bpr_I1862	C	3.7×10^{-14}	5.0	42.9	27%
DNA polymerase III β-subunit, DnaN	Bpr_I0002	C	5.1×10^{-12}	4.7	41.4	25%
FtsK/SpoIIIE family protein	Bpr_I1301	M	6.7×10^{-5}	5.0	105.3	5%
Single-stranded DNA binding protein, Ssb1	Bpr_I0240	C	2.2×10^{-14}	4.7	16.6	27%
**Cell Envelope**						
Aminotransferase DegT/DnrJ/EryC1/StrS family	Bpr_I0345	C	1.1×10^{-16}	5.8	44.9	31%
Aminotransferase DegT/DnrJ/EryC1/StrS family	Bpr_I2543	C	1.9×10^{-11}	5.1	47.3	11%
dTDP-4-dehydrorhamnose 3,5-epimerase, RfbC2	Bpr_I0548	C	2.7×10^{-8}	4.7	20.6	14%
dTDP-4-dehydrorhamnose reductase, RfbD1	Bpr_I2582	C	4.5×10^{-10}	4.8	32.1	15%
dTDP-glucose 4,6-dehydratase, RfbB	Bpr_I0545	C	2.2×10^{-11}	5.5	44.1	20%
Glycosyl transferase GT28 family	Bpr_I2555	C	9.5×10^{-7}	4.9	40.1	9%
NAD dependent epimerase/dehydratase	Bpr_I0348	C	3.2×10^{-12}	5.9	38.9	17%
NAD dependent epimerase/dehydratase	Bpr_I2647	C	2.1×10^{-10}	5.5	38.8	8%
NAD-dependent epimerase/dehydratase	Bpr_I0517	C	5.4×10^{-14}	4.9	42.0	9%
NAD-dependent epimerase/dehydratase	Bpr_I2537	C	1.1×10^{-15}	4.7	36.0	23%
Nucleotide sugar dehydrogenase	Bpr_I0828	C	2.6×10^{-11}	5.0	46.1	11%
Nucleotide sugar dehydrogenase	Bpr_I2538	C	1.8×10^{-9}	4.9	48.6	6%
Oxidoreductase GFO/IDH/MOCA family	Bpr_I0406	C	2.2×10^{-12}	5.6	40.5	9%
Phosphoglucomutase/phosphomannomutase family protein	Bpr_I0554	C	1.0×10^{-30}	4.7	66.0	38%
Polysaccharide biosynthesis protein	Bpr_I2562	C	5.3×10^{-14}	5.1	43.6	23%
Rod shape-determining protein, MreB1	Bpr_I1486	C	1.1×10^{-15}	5.5	36.6	20%
UDP-galactopyranose mutase, Glf	Bpr_I2305	C	3.6×10^{-11}	4.8	46.2	9%
**Cellular Processes**						
Flagellin, FliC1	Bpr_I0488	C	1.7×10^{-10}	5.2	31.4	27%
Flagellin, FliC2	Bpr_I0489	C	1.0×10^{-30}	5.2	30.8	28%
Flavin reductase domain-containing protein	Bpr_I0363	C	7.5×10^{-11}	5.1	23.4	18%
Rubrerythrin, Rbr1	Bpr_I0031	C	3.4×10^{-9}	5.1	19.7	34%
Rubrerythrin, Rbr2	Bpr_I0362	C	1.6×10^{-12}	4.9	21.3	34%
Superoxide dismutase, SodA	Bpr_I0467	C	1.0×10^{-30}	5.3	24.1	67%
**Central Metabolism**						
Carboxynorspermidine dehydrogenase	Bpr_I1198	C	2.9×10^{-11}	4.6	47.4	9%
Formate-tetrahydrofolate ligase, Fhs	Bpr_I1302	C	1.0×10^{-30}	6.0	60.0	24%
S-adenosylmethionine synthetase, MetK	Bpr_I2769	C	1.9×10^{-13}	4.9	43.1	57%
**Energy Metabolism**						
3-hydroxybutyryl-CoA dehydrogenase, Hbd	Bpr_I2486	S	1.0×10^{-30}	5.2	31.2	71%
Acetate kinase, AckA	Bpr_I1580	C	7.0×10^{-13}	5.9	42.7	29%
Acetyl-CoA synthetase, AscA	Bpr_I1274	C	2.3×10^{-13}	6.0	64.9	9%
Aconitate hydratase, AcnA	Bpr_I2797	C	5.9×10^{-10}	5.3	82.7	8%
ATP synthase F1 α-subunit, AtpA1	Bpr_I0166	C	1.9×10^{-12}	5.3	55.0	27%
ATP synthase F1 β-subunit, AtpD1	Bpr_I0168	C	6.9×10^{-13}	4.6	50.3	45%
ATP synthase F1 β-subunit, AtpD2	Bpr_I1162	C	3.0×10^{-8}	4.5	50.9	16%
Butyrate kinase, Buk	Bpr_I2323	C	9.9×10^{-15}	5.1	38.7	74%

Table S2. *Cont.*

Protein	Locus	Location	Score	p*I*	kDa	Cov.
**Energy Metabolism**						
Butyryl-CoA dehydrogenase, Bcd	Bpr_I2485	C	1.0×10^{-30}	6.1	42.5	73%
Crotonase, Crt	Bpr_I2487	C	1.0×10^{-30}	4.7	28.0	55%
Electron transfer flavoprotein α-subunit, EtfA	Bpr_I2483	C	1.0×10^{-30}	4.8	37.4	46%
Electron transfer flavoprotein β-subunit, EtfB	Bpr_I2484	C	2.2×10^{-15}	5.2	28.4	44%
Iron-containing alcohol dehydrogenase	Bpr_I1425	C	1.0×10^{-30}	5.1	44.4	52%
Isocitrate dehydrogenase, Icd	Bpr_I1102	C	1.1×10^{-15}	5.1	45.1	16%
Malate dehydrogenase, Mdh	Bpr_I2277	C	2.4×10^{-9}	4.8	41.4	9%
Methylmalonyl-CoA decarboxylase α-subunit, MmdA	Bpr_I1226	C	5.3×10^{-14}	4.5	50.9	24%
Oxaloacetate decarboxylase α-subunit, OadA	Bpr_I1230	C	1.1×10^{-15}	5.2	52.4	18%
Phosphate acetyltransferase, Pta	Bpr_III010	C	2.2×10^{-16}	4.7	22.3	32%
Phosphate acetyltransferase, Pta	Bpr_III011	C	7.9×10^{-13}	5.2	14.4	59%
Phosphate butyryltransferase, Ptb	Bpr_I2324	C	5.6×10^{-16}	5.1	33.3	57%
Pyruvate carboxyltransferase domain-containing protein	Bpr_I1100	C	5.4×10^{-5}	5.4	57.2	7%
Pyruvate formate lyase, PflB	Bpr_I0112	C	1.0×10^{-30}	5.7	84.9	65%
Pyruvate phosphate dikinase, PpdK	Bpr_I1154	C	1.0×10^{-30}	4.8	104.3	52%
Pyruvate:ferredoxin oxidoreductase	Bpr_I0269	C	1.0×10^{-30}	5.2	127.3	48%
Thiolase, ThlA1	Bpr_I2488	C	1.0×10^{-30}	5.0	42.0	68%
Thiolase, ThlA2	Bpr_I2475	S	1.0×10^{-30}	6.0	41.0	66%
**Hypothetical**						
Hypothetical protein	Bpr_I0212	C	1.1×10^{-16}	5.2	41.6	28%
Hypothetical protein	Bpr_I0219	S	8.9×10^{-11}	3.9	30.3	5%
Hypothetical protein	Bpr_I0280	C	2.4×10^{-11}	8.4	23.2	15%
Hypothetical protein	Bpr_I0351	C	3.6×10^{-14}	5.0	34.4	6%
Hypothetical protein	Bpr_I0566	M	5.4×10^{-9}	9.9	28.0	5%
Hypothetical protein	Bpr_I0799	C	1.9×10^{-8}	4.1	13.5	14%
Hypothetical protein	Bpr_I0801	C	1.1×10^{-11}	4.3	23.4	21%
Hypothetical protein	Bpr_I0874	C	2.1×10^{-10}	4.5	14.8	13%
Hypothetical protein	Bpr_I0876	S	2.0×10^{-4}	4.2	63.1	3%
Hypothetical protein	Bpr_I0952	C	2.2×10^{-15}	3.8	37.2	26%
Hypothetical protein	Bpr_I1041	C	8.1×10^{-12}	4.1	9.3	39%
Hypothetical protein	Bpr_I1177	C	3.6×10^{-13}	4.3	24.7	9%
Hypothetical protein	Bpr_I1179	C	1.0×10^{-30}	4.7	24.9	51%
Hypothetical protein	Bpr_I1180	C	1.0×10^{-30}	7.4	36.4	66%
Hypothetical protein	Bpr_I1300	C	3.6×10^{-12}	4.7	21.8	50%
Hypothetical protein	Bpr_I1457	C	3.8×10^{-9}	4.2	25.9	18%
Hypothetical protein	Bpr_I1473	C	1.8×10^{-10}	5.4	24.1	18%
Hypothetical protein	Bpr_I1477	C	3.5×10^{-12}	4.2	19.1	29%
Hypothetical protein	Bpr_I1479					
